# Emerging Roles of Exocyst Complex in Fungi: A Review

**DOI:** 10.3390/jof10090614

**Published:** 2024-08-28

**Authors:** Qussai Zuriegat, Yakubu Saddeeq Abubakar, Zonghua Wang, Meilian Chen, Jun Zhang

**Affiliations:** 1 State Key Laboratory of Ecological Pest Control for Fujian and Taiwan Crops, Ministerial and Provincial Joint Innovation Centre for Safety Production of Cross-Strait Crops, College of Plant Protection, Fujian Agriculture and Forestry University, Fuzhou 350002, China; qussai.zurriegat@gmail.com (Q.Z.); ay.saddeeq@yahoo.com (Y.S.A.); zonghuaw@163.com (Z.W.); 2 Fujian Key Laboratory on Conservation and Sustainable Utilization of Marine Biodiversity, College of Materials and Chemical Engineering, Minjiang University, Fuzhou 350108, China

**Keywords:** exocyst complex, vesicle trafficking, fungal pathogenicity, polarized growth, host-fungal interactions

## Abstract

The exocyst complex, an evolutionarily conserved octameric protein assembly, plays a central role in the targeted binding and fusion of vesicles at the plasma membrane. In fungal cells, this transport system is essential for polarized growth, morphogenesis, cell wall maintenance and virulence. Recent advances have greatly improved our understanding of the role and regulation of the exocyst complex in fungi. This review synthesizes these developments and focuses on the intricate interplay between the exocyst complex, specific fungal cargos and regulatory proteins. Insights into thestructure of the exocyst and its functional dynamics have revealed new dimensions of its architecture and its interactions with the cellular environment. Furthermore, the regulation of exocyst activity involves complex signaling pathways and interactions with cytoskeletal elements that are crucial for its role in vesicle trafficking. By exploring these emerging themes, this review provides a comprehensive overview of the multifaceted functions of the exocyst complex in fungal biology. Understanding these mechanisms offers potential avenues for novel therapeutic strategies against fungal pathogens and insights into the general principles of vesicle trafficking in eukaryotic cells. The review therefore highlights the importance of the exocyst complex in maintaining cellular functions and its broader implications in fungal pathogenicity and cell biology.

## 1. Introduction

Vesicle trafficking is a fundamental process in eukaryotic cells, essential for the transport of proteins, lipids, and other cellular components between different membrane-bound organelles [1,2]. In fungi, this intricate system plays a crucial role in various biological processes, including growth, development, and response to environmental cues [3]. One key player in the regulation of vesicle trafficking, particularly in the final step of secretion, is the exocyst complex. The exocyst is an evolutionarily conserved octameric protein complex that acts as a tethering factor, facilitating the precise docking and fusion of secretory vesicles with the plasma membrane [4]. Its intricate architecture and interaction with regulatory proteins enable spatial and temporal control over exocytosis, ensuring the accurate delivery of cargo molecules to their destination. Initially identified in the budding yeast *S. cerevisiae*, the exocyst complex has been discovered in diverse organisms, including plants, fungi and mammals, where it plays pivotal roles in cell polarity, cytokinesis and exocytosis [5]. In fungi, the exocyst complex assumes essential functions in growth, development and pathogenesis. The exocyst complex ensures precise spatial control during the tethering of exocytic vesicles by interacting with proteins and lipids present on both the vesicle and the plasma membrane. It facilitates the precise targeting and fusion of vesicles at the plasma membrane, critical for fungal morphogenesis and the secretion of virulence factors during infection [6,7,8]. Dysfunction or disruption of the exocyst complex in fungi has been linked to various diseases, underscoring its importance in fungal biology and pathogenicity. 

In recent years, significant advances have been made in understanding the structure, function and regulation of the exocyst complex in fungi [9,10]. These studies have revealed the exocyst complex as a dynamic and multifaceted machinery that orchestrates the secretion of enzymes, toxins and other effectors, enabling fungi to adapt to changing environments and interact with their hosts. The exocyst complex has also been shown to play a crucial role in fungal cell wall and septum formation, as well as in hyphal growth [11,12,13,14,15,16,17]. While the core components of the exocyst complex are generally well-conserved, emerging evidence suggests that its specific functions and regulatory mechanisms may vary among organisms. This variability implies that the exocyst may not function as a single biochemical entity across all species. Studies across various organisms, including fungi, plants and animals, have revealed species-specific differences in the composition, regulation and physiological roles of the exocyst complex [18,19,20,21]. Genetic analyses have provided invaluable insights into the physiological roles and regulatory mechanisms of the exocyst complex in filamentous fungi [13,14].

The localization of the exocyst complex indeed shows variations between yeast and filamentous fungi. In yeast, such as *S. cerevisiae*, the exocyst complex localizes primarily to sites of active exocytosis at the plasma membrane, where it mediates the tethering of secretory vesicles before fusion. This localization is typically observed at the bud tip during budding yeast cell division [22]. In contrast, filamentous fungi, including species like *Aspergillus nidulans*, *N. crassa* and *F. odoratissimum*, display more diverse and dynamic localization patterns of the exocyst complex. These fungi have a more complex morphology with elongated hyphae (filaments), and the exocyst complex can localize to various sites along the hyphae, depending on the stage of growth and development. This localization includes growing hyphal tips, subapical regions, septa (cross-walls between hyphal compartments), and even regions undergoing branching or specialized growth [13,14,15,16,17]. Furthermore, in filamentous fungi, the exocyst complex may play additional roles beyond conventional exocytosis, such as in the delivery of cell wall material during hyphal growth and in the establishment of polarity and cell shape. These differences reflect the diverse functions and adaptations of the exocyst complex in different fungal species and highlight the flexibility of its localization patterns in response to specific growth and developmental requirements of each organism.

Beyond its role in vesicle trafficking, the exocyst complex is involved in various cellular functions, including cytokinesis, cell polarization, migration, invasion, cytoplasmic division, primary ciliogenesis and autophagy [5,6]. Recent structural analyses of exocyst subunits and complexes, along with studies on their dynamic assembly, have broaden our understanding of the mechanisms underlying exocyst functions [23,24].

Despite extensive genetic analyses and the observation of fungi-specific phenotypes in exocyst complex mutants, direct evidence linking these phenotypes to exocyst function remains elusive. While studies have provided compelling correlations between exocyst mutations and various cellular dysfunctions, such as impaired polarized growth, altered cell wall composition, and reduced virulence in pathogens, definitive proof of the exocyst’s precise mechanisms and interactions in these processes is yet to be established [13,14,15,16,17]. One of the challenges lies in the complexity of the exocyst complex itself and its intricate regulatory networks within fungal cells. Although genetic studies have identified mutations that affect exocyst assembly or function, teasing apart the specific contributions of the exocyst to each observed phenotype requires more targeted experimental approaches. These could include advanced imaging techniques to visualize vesicle trafficking dynamics in real-time, biochemical assays to dissect protein-protein interactions within the exocyst complex, or the development of novel genetic tools to manipulate exocyst components with greater precision. Moreover, while genetic analyses are invaluable for generating hypotheses and exploring potential functions of the exocyst, they often rely on indirect evidence such as phenotypic correlations and pathway interactions. Direct evidence would ideally involve demonstrating the physical interaction of the exocyst with specific cargo molecules or regulatory factors, directly observing exocyst-mediated vesicle fusion events at the plasma membrane, or manipulating exocyst activity to rescue or exacerbate specific phenotypes.

In conclusion, while genetic analyses have provided a wealth of insights into the role of the exocyst complex in fungi, the field awaits more direct evidence to firmly establish how this complex orchestrates vesicle trafficking and contributes to fungal physiology and pathogenesis. Future research efforts aimed at bridging this gap will be essential for deepening our understanding of exocyst function and its potential as a target for therapeutic interventions in fungal diseases. This review aims to provide a comprehensive overview of the recent advancements made in the structure and functions of the exocyst complex, put forward some potential antifungal therapies and biotechnological applications and outline future perspectives for better understanding of the complex particularly in fungi.

### 1.1. Overview of the Exocyst Complex

A recent phylogenetic study of the exocyst has highlighted that the complex is present in most major eukaryotic lineages, including fungi, animals, plants and several protist lineages, with the exception of Alveolata [20,25]. In addition, almost all lineages containing the exocyst have homologues of the eight exocyst subunits. This suggests that the exocyst is an ancient and important complex that plays a key role in polarized exocytosis, which is the secretion of vesicle cargo to a specific site on the plasma membrane.The exocyst also plays a role in late stages of endocytosis, cell division, cell migration and cell signaling [5]. The late stages of endocytosis involve the re-internalization of plasma membrane components, which is essential for maintaining membrane integrity and often occurs at the site where exocytosis previously took place. Infungi, exocyst function is essential for viability and polarized growth [4,13,15]. Studies have provided evidence that the exocyst is essential for the establishment and maintenance of cell polarity. In *S. cerevisiae*, exocyst mutants are defective in polarized secretion and often exhibit morphologies characteristic of loss of cell polarity [4]. Loss of cell polarity is usually associated with cancer and tumor development in animals, which highlights the importance of understanding exocyst function and the underlying molecular mechanisms [26,27]. Aberrant cell polarity has also been associated with invasive growth of some fungi, implying that research into the polarized growth and its regulation may be of great importance. Exocyst complex has recently been the focus of many studies in the budding yeast *S. cerevisiae* and other filamentous fungi [14,15,16,28].

### 1.2. Importance of the Exocyst Complex in Fungi

Exocytosis is an important process in all eukaryotic cells. In fungi, it is essential for polarized growth, spore formation, and response to the environment. Gene knockouts of the exocyst components have varied effects depending on the organism and the conditions involved [13,14,15,16,17,29]. In *S. cerevisiae*, the best studied fungal organism, germinating spores use stored lipids and amino acids to produce polarized or germ tubes [30]. The growth of this tube requires exocytosis to deliver vesicles containing cell wall materials and membranes to the growing tip. A conditional mutant of *SEC3*, which is a component of the exocyst, shows a deposition of cell wall materials around the arrest site. This implies that without the ability to tether exocytic vesicles, such materials will accumulate at the delivery site [11,28,31,32]. In *S. cerevisiae*, it is well established that polarized growth requires localized exocytosis [8,33,34]. This is also apparent from studies on germ tubes in *Magnaporthe oryzea* and *N. crassa*, two phytopathogenic fungi that use different strategies to produce infectious structures [15,16]. *M. orysea* uses turgor pressure to develop a penetration peg forcefully rupture and penetrate the host tissues [16]. Spitzenkörper, a dense accumulation of vesicles and cytoskeletal elements, thought to be involved in the establishment of polarity, growth and reorientation processes (reviewed by Howard and Valent, 1996 [35]), is formed at the growing tip of the peg. The Spitzenkörper is a feature of all fungi that grow using polarized apical extension and is thought to be analogous to the growing tip of a plant or animal cell [7]. Studies on Spitzenkörper in the basidiomycete *Coprinopsis cinerea* revealed that it is enriched with secretory vesicles. A temperature-sensitive mutant of an exocyst component in *C. cinerea* developed hyperbranched hyphae and abnormal appressoria, defects similar to loss of *EXO70* in *Magnaporthe oryzae* [16,36]. This, and future studies on this area, would further our understanding of the significance of the exocyst complex in polarized growth and morphogenesis, and unveil some novel therapeutic strategies for targeting fungal pathogens.

## 2. Structural Composition of the Exocyst Complex in Fungi

The exocyst complex is a cornerstone of the cellular machinery in fungi that orchestrates the precise delivery of vesicles to the plasma membrane. The exocyst consists of a conserved assembly of protein subunits and plays a central role in various cellular processes, including polarized growth, secretion and pathogenesis [37]. Recent structural studies have provided detailed insights into its architecture and functional domains, emphasizing its role in tethering vesicles to specific sites on the plasma membrane prior to fusion [23]. Regulation of the exocyst complex involves interactions with Rho GTPases, such as Rho1 and Cdc42, which modulate its localization and activity [38]. These interactions are critical for the spatial and temporal control of exocytosis, ensuring precise delivery of cargo to the cell periphery [6]. Furthermore, the evolutionary conservation of the exocyst complex underscores its importance across fungal species, despite variations in sequence and regulatory mechanisms [5]. This section provides an in-depth analysis of the molecular architecture of the exocyst, examining the individual subunits and their contributions to facilitating the processes of vesicle tethering and fusion. Understanding these structural and regulatory aspects not only enhances our knowledge of fundamental cellular processes but also holds potential implications for therapeutic strategies targeting fungal infections [39].

### 2.1. Core Components of the Exocyst Complex

The exocyst complex plays a crucial role in facilitating vesicle trafficking and secretion within cells. Consisting of eight core subunits—Sec3, Sec5, Sec6, Sec8, Sec10, Sec15, Exo70 and Exo84 [11]—this complex was first identified in yeast as a molecular machine with a total molecular weight of approximately 845 kDa (see Table 1). It plays a key role in regulating the exocytosis of secretory vesicles [11,37,40]. Numerous studies have shown that the exocyst complex binds to the secretory vesicle through interactions with Sec10 and Sec15 [41,42,43], while Sec3 and Exo70 are responsible for binding to exocytic sites on the plasma membrane [34,44,45,46,47,48]. While it was initially characterized in model organisms such as yeast and mammals, more recent studies have elucidated the structural organization of the exocyst complex in fungi, shedding light on both conserved features and unique adaptations across different species [11,40,49,50]. The exocyst complex forms a multi-subunit scaffold that mediates the tethering and fusion of vesicles at specific sites on the plasma membrane during exocytosis [5,23,51,52]. Structural studies demonstrated the organization and architecture of the exocyst complex, revealing its intricate design and subunit arrangement. Both cryo-electron microscopy (cryo-EM) and X-ray crystallography revealed that the exocyst complex assumes a horseshoe-shaped structure. Furthermore, all the subunits exhibit a uniform structural organization and associate to form an elongated structure measuring 32 nm in length and 13 nm in width. This structure categorizes the exocyst complex as a member of the Complex Associated with Tethering Containing Helical Rods (CATCHRs) protein family, similar to related complexes such as the Conserved Oligomeric Golgi complex (COG) and the Golgi-Associated Retrograde Protein complex (GARP) [10,23,53]. This structure resembles a boomerang, with two distinct subcomplexes: subcomplex I and subcomplex II [23]. SubcomplexI comprises Sec3, Sec5, Sec6 and Sec8, while subcomplex II consists of Sec10, Sec15, Exo70 and Exo84. The interactions between these subcomplexes are critical for the assembly and function of the exocyst complex. Subcomplex I and subcomplex II interact with each other to form a holo-complex, and this interaction is predominantly mediated by Sec8 and Sec5 from subcomplex I. Generally, the various interactions are facilitated by extensive binding interfaces along the rod-like structures of Sec8 and Sec5, allowing them to bind to multiple subunits of the subcomplex II [23,54,55]. Similar to animals, each exocyst subunit in fungi is typically encoded by a single gene. These genes play distinct roles in facilitating vesicle trafficking and ensuring the precise delivery of cargo to the plasma membrane [23,54]. The intricate interactions and coordination among these subunits, as well as their dynamic association with various regulatory proteins, are essential for the exocyst’s diverse cellular functions [5,24,56]. In recent years, significant progress has been made in elucidating the structural organization and functional mechanisms of the exocyst complex. Cryo-electron microscopy studies have provided detailed insights into the architecture of the exocyst, revealing its remarkable plasticity and flexibility, which are crucial for its role as a spatial and temporal regulator of vesicle trafficking [57] (unpublished manuscript). Moreover, the exocyst has been found to interact with a multitude of other key players in the cellular trafficking machinery, such as Rab GTPases, Rho GTPases, SNARE proteins and Sec1/Munc18 (SM) regulators, thereby coordinating various aspects of membrane fusion and cargo delivery [24,58,59,60].

### 2.2. Interactions among the Exocyst Complex Subunits

The exocyst complex has been thoroughly investigated in *S. cerevisiae*. However, even among the researchers studying the budding yeast, there is considerable debate about the structure and function of this complex. It was initially postulated that the assembly of the exocyst complex takes place in a sequential process. Recent studies have uncovered a complex network of interactions between the exocyst subunits in fungi and shed light on their molecular mechanisms and functional significance [45,48,51,71]. The interaction between Sec3 and Exo70 has been shown to be a key step in the assembly of the exocyst complex, facilitating its localization to specific sites on the plasma membrane and controlling the processes of vesicle targeting and fusion processes [34]. He et al. (2007) demonstrated the direct interaction between Sec3 and Exo70, which is crucial for the formation of the exocyst complex, while Zhang et al. (2008) emphasized its importance in the spatial regulation of exocytosis. Subsequent studies unveiled the dynamic nature of the Sec3-Exo70 interaction, revealing its regulatory mechanisms and functional significance [51,72]. Live-cell imaging techniques have provided real-time visualization of this interaction, corroborating its role in cellular processes such as cell polarization and tissue morphogenesis [37,73].

Similarly, the interaction between Sec6 and Sec8 forms a stable subcomplex essential for exocyst complex assembly and function, regulating vesicle targeting and fusion during exocytosis. Guo et al. (1999) and Hsu et al. (1998) highlighted the critical role of the Sec6–Sec8 interaction in this process, with subsequent structural and functional studies elucidating its regulatory mechanisms and functional significance [70,74]. Genetic and model organism studies further affirmed the essentiality of the Sec6–Sec8 interaction in cellular processes such as cell polarization and cytokinesis [73]. The Sec10–Sec15 interaction stabilizes the exocyst complex, facilitating vesicle tethering and fusion during exocytosis. Hutagalung et al. (2009) demonstrated the direct interaction between Sec10 and Sec15, essential for complex stability, with subsequent studies revealing its structural features and spatiotemporal regulation [23,75]. Functional studies corroborated the significance of the Sec10–Sec15 interaction in cellular processes such as cell polarization and tissue morphogenesis [73,76]. The interactions among Sec5, Sec10, and Sec15 are characterized by their flexibility and dynamic nature. Sec5 is known to form a transient complex with Sec10 and Sec15, thereby undergoing significant conformational changes during vesicle tethering process. These dynamic interactions are critical for the adaptability of the exocyst complex in response to various cellular signals and demands [24]. Lastly, the interaction between Sec5 and Exo84 regulates exocytosis and coordinates the functioning of the exocyst complex. Zhang et al. (2001) and Boyd et al. (2004) elucidated the direct interaction between Sec5 and Exo84, which is essential for the exocyst complex assembly and spatial regulation of exocytosis. Structural and functional studies provided insights into the regulatory mechanisms and functional significance of the exocyst complex [45,77], which demonstrated its importance in cellular processes like cell polarization and cytokinesis [78]. The specific interactions among the exocyst subunits are essential for the involvement of the complex in vesicle trafficking and cell polarity. These interactions ensure accurate tethering and fusion of the vesicles at specific sites on the plasma membrane, which is crucial for processes like cytokinesis, bud formation and polarized growth. Disruptions of these interactions can lead to defects in exocytosis and other cellular processes, underscoring the importance of these molecular interactions in cell viability and function [4]. Interactions among the exocyst complex subunits in fungi are intricate and highly regulated, involving specific and dynamic associations that are crucial for the functions of the complex. Recent advances in structural biology and molecular studies have significantly enhanced our understanding of these interactions, providing a detailed picture of how the exocyst complex coordinates vesicle trafficking and membrane dynamics [23,24]. This knowledge is fundamental for elucidating the broader mechanisms of cellular organization and function in fungi.

### 2.3. Interactions of the Exocyst Complex with Regulatory Proteins

The exocyst complex does not function in isolation; it interacts with some regulatory proteins to coordinate vesicle trafficking. In the fungus *Magnaporthe oryzae*, the causal agent of rice blast disease, septins found to play a crucial role in coordinating the interaction with the exocyst complex during fungal invasion. These GTP-binding proteins form a lateral diffusion barrier at the appressorium pore, ensuring the correct localization of the exocyst octameric complex. This positioning is vital for polarized exocytosis, where secretory vesicles carrying effector proteins are delivered to the site of infection, facilitating the emergence of the penetration peg, a structure that breaches the host plant cell wall [16].The interaction between septins and the exocyst is mediated through key cellular regulators, such as Rho GTPases (Rho1 and Rac1), which control cytoskeletal organization and exocytosis. Co-immunoprecipitation studies have shown that septins directly interact with exocyst components Sec6 and Exo84, along with Rho GTPases and fimbrin, coordinating the actin cytoskeleton for precise vesicle trafficking to the infection site. This interaction helps maintain the polarized structure of the appressorium, essential for effective host invasion.Septins also ensure the polarized secretion of effector proteins, which are involved in suppressing plant immune responses. These effectors are likely secreted at the base of the appressorium at the onset of penetration peg formation, a process that requires the coordinated activity of septins and the exocyst [79]. By maintaining the appressorium’s polarized architecture and coordinating vesicle trafficking, septins ensure the precise delivery of effectors, critical for the pathogen’s success in invading the host.

Rho GTPases, such as Rho1p and Cdc42p in fungi, play significant roles in regulating exocyst localization and activity. These small GTPases interact with exocyst subunits like Sec3 and Exo70, targeting the exocyst to sites of active growth such as the budding site in yeast. These interactions ensure that vesicles are delivered to precise cellular locations, which is essential for maintaining cell polarity and growth [77,80,81]. Rab GTPases are also critical regulators of the exocyst complex. In *S. cerevisiae*, the Rab GTPase Sec4p interacts with the exocyst subunit Sec15p, facilitating the tethering of secretory vesicles to the plasma membrane. This interaction is regulated by the GTPase activity of Sec4p, which controls the recruitment of the exocyst complex to vesicles, ensuring precise timing and localization of vesicle fusion events [70]. This regulatory mechanism highlights the importance of GTPase activity in modulating exocyst function and vesicle trafficking. SNARE proteins are indispensable for the final step of vesicle fusion with target membranes. The exocyst complex facilitates the assembly of SNARE complexes by bringing vesicles close to the plasma membrane. In fungi, the exocyst subunit Sec6 interacts with the t-SNARE protein Sso1p, promoting the formation of the SNARE complex necessary for vesicle fusion. This interaction is essential for efficient exocytosis and is tightly regulated to ensure that vesicle fusion occurs only at specific plasma membrane sites [65]. Sec1/Munc18 (SM) proteins are essential for SNARE-mediated membrane fusion, acting as chaperones that facilitate the assembly and function of SNARE complexes. In yeast, the exocyst subunit Sec1 interacts with the exocyst complex to coordinate the docking and fusion of secretory vesicles. This interaction stabilizes the exocyst-SNARE complex assembly, ensuring efficient vesicle fusion [82]. Precise regulation of this interaction is crucial for maintaining the fidelity of vesicle trafficking and membrane fusion processes. Post-translational modifications, such as phosphorylation and dephosphorylation of exocyst subunits, are critical regulatory mechanisms that modulate the function of the exocyst complex. Various kinases and phosphatases interact with the exocyst complex to regulate its activity. For instance, the yeast kinase Pkc1 phosphorylates the exocyst subunit Exo70, modulating its interaction with the plasma membrane and other exocyst subunits. This phosphorylation event is crucial for the spatial regulation of exocyst function, ensuring that vesicle fusion occurs at correct cellular locations [83]. The actin cytoskeleton plays a significant role in the localization and function of the exocyst complex. Actin filaments provide tracks for the transport of secretory vesicles to the plasma membrane, and the exocyst complex interacts with actin-regulating proteins to coordinate this process. In yeast, the actin-binding protein Abp1 interacts with the exocyst subunit Sec3, linking the exocyst complex to the actin cytoskeleton and facilitating vesicle transport. This interaction is essential for maintaining the directionality and efficiency of vesicle trafficking [84]. Cell cycle regulators also interact with the exocyst complex to coordinate vesicle trafficking with cell cycle progression. In *S. cerevisiae*, the cyclin-dependent kinase Cdk1 phosphorylates multiple exocyst subunits, including Sec5 and Sec8, during the cell cycle. These phosphorylation events regulate the assembly and function of the exocyst complex, ensuring that vesicle trafficking is coordinated with cell division and other cell cycle processes. This coordination is crucial for maintaining cellular homeostasis and ensuring proper cell growth and division [85]. The interactions between the exocyst complex and regulatory proteins in fungi are highly intricate and involve multiple layers of regulation. These interactions ensure the precise coordination of vesicle trafficking, cell polarity, and membrane fusion, which are essential for various cellular processes. Recent advances in molecular and structural biology have provided significant insights into these interactions, highlighting their importance in maintaining cellular homeostasis and responding to environmental cues.

## 3. Comparison of the Exocyst Complex in Fungi and Other Model Organisms

The Exocyst complex’s core structure is highly conserved across fungi, mammals and plants, emphasizing its essential role in fundamental cellular processes. However, its localization and function are adapted to the unique cellular and physiological needs of each organism. These adaptations enable the Exocyst complex to support a wide range of processes, from polarized growth in fungi to complex secretion and cell division in mammals, and targeted growth in plants. Understanding these differences not only highlights the versatility and indispensability of the complex but also provides insights into the evolutionary adaptations that have allowed diverse eukaryotic lineages to thrive.

### 3.1. Comparison of Exocyst Structure in Fungi and Model Organisms

The exocyst complex serves as a fundamental regulator of vesicle trafficking and membrane fusion events in eukaryotic cells, playing pivotal roles in cellular processes such as polarized growth, secretion and organelle biogenesis. The exocyst, which consists of evolutionarily conserved protein subunits, is an intricate molecular machine that is essential for maintaining cellular homeostasis and facilitating diverse physiological functions [5,20,49,86,87,88,89]. Recent studies of the exocyst complex shed light on its structural organization across organisms. While mammals and fungi typically possess an octameric exocyst complex for late-stage exocytosis, recent genomic studies suggest that trypanosomes deviate from this and may contain only six subunits. However, analysis of the *Trypanosoma brucei* exocyst complex revealed it to be nonameric, with all eight canonical subunits present despite the sequence differences. Additionally, a new essential subunit, Exo99, was identified, enhancing our understanding of membrane transport in *Trypanosoma brucei*, a protist responsible for African trypanosomiasis [20,25]. This discovery offers insights into pathogenesis mechanisms and potential therapeutic targets. In the realm of plant biology, comparative genomics studies have revealed intriguing insights into the diversity of the exocyst complex. Unlike in animals and fungi, in land plants, with the exception of SEC6 and SEC8, exocyst subunits are usually duplicated or even multiplied, with an extreme evolutionary proliferation of isoforms observed in the case of the EXO70 subunit [90,91]. These isoforms play distinct roles in plant cell biology, influencing processes such as cell wall synthesis and growth regulation. These revelations prompt a reevaluation of the structural organization and functional dynamics of the exocyst complex across diverse organisms, transcending traditional boundaries and fostering interdisciplinary collaborations. The integration of cutting-edge technologies, such as cryo-electron microscopy and proximity labeling, holds promise for elucidating the intricate architecture and regulatory networks governing exocyst-mediated membrane trafficking events. By unraveling the structural nuances and functional adaptations of the exocyst complex, researchers can decipher fundamental principles underlying cellular organization and disease pathogenesis, paving the way for innovative therapeutic strategies and biomedical interventions.

### 3.2. Comparing Exocyst Localization in Fungi and Other Model Organisms

Recent studies revealed the diverse spatial distributions and regulatory mechanisms of the exocyst complex, consistent with the dynamic nature of membrane trafficking processes. These adaptations cater to distinct cellular processes and environmental cues, underscoring the exocyst complex’s varied functions in cellular physiology and development across species. In fungi, the exocyst complex is crucial for polarized growth and cell wall synthesis, essential for morphogenesis and nutrient uptake. For instance, in *S. cerevisiae*, fluorescence microscopy studies have demonstrated the concentrated localization of exocyst subunits at the bud neck during cell division [28,34,45]. In filamentous fungi, such as *N. crassa*, *Ashbya gossypii*, *M. oryzae*, *Aspergillus oryzae* and *F. odoratissimum*, exocyst subunits predominantly localize at the hyphal tips, though diverse patterns have been observed [13,14,15,16,17]. Notably, in *A. gossypii*, the localization of specific exocyst subunits varies between slow and fast-growing hyphae [13]. In *A. nidulans* and *C. albicans*, the exocyst complex localizes anterior to the Spitzenkörper and within a crescent tip structure, respectively, highlighting its role in polarized growth and secretion [92,93]. Additionally, in *F. odoratissimum*, exocyst subunits are found not only at the growing tips but also at the outer edge of septa in mature hyphae, suggesting their involvement in septum-directed protein secretion [17]. While cellular expansion primarily occurs at the hyphal tip in filamentous fungi, studies in *Aspergillus niger* and *A. oryzae* have shown that exocytosis can also occur subapically, particularly for certain hydrolytic enzymes secreted from septa [14,94]. Specifically, in *A. oryzae*, the exocyst subunit Sec3 has been implicated in α-amylase secretion at the septum [14]. Additionally, a recent study in *F. odoratissimum* found that Exo70 and Sec5 are required for septum-directed secretion of α-amylase, although they perform different roles in this process [17]. In higher eukaryotes, such as humans, the exocyst complex displays diverse subcellular localization patterns [5,54]. In mammalian cells, exocyst components localize to the leading edge of migrating cells, the cleavage furrow during cytokinesis, and the neuronal growth cone during axonal outgrowth [95,96]. For instance, in migrating cells, the exocyst localizes to the leading edge, promoting the secretion of matrix metalloproteinases involved in extracellular matrix remodeling [68]. In T cells, it concentrates at the immunological synapse during antigen presentation, facilitating the polarized secretion of cytokines and cytolytic granules [97]. These spatially regulated dynamics are crucial for immune cell activation, cell migration and neuronal polarization. In plants, the exocyst complex is vital for cell wall deposition, pollen tube growth, and root hair elongation. For example, in *Arabidopsis thaliana*, exocyst components localize to the growing tips of root hairs and pollen tubes, mediating the secretion of cell wall components and promoting cell expansion [90]. The exocyst is also involved in the formation of plasmodesmata, specialized channels connecting adjacent plant cells for nutrient and signal exchange [98].

## 4. Regulation Mechanisms of the Exocyst Complex in Fungi

The regulation of exocyst complex in fungi is a highly coordinated process that involves the assembly, localization and function of the complex. This complex serves as a pivotal regulator of vesicle trafficking and fusion with the plasma membrane in fungi, ensuring precise spatial and temporal control of cellular processes crucial for growth, development and pathogenesis. The assembly of exocyst complex begins with the synthesis of individual subunits, followed by their transport to the plasma membrane. The subunits then interact with each other, leading to the formation of the complete exocyst complex. Several regulatory factors including phosphorylation and post-translational modifications, influence the assembly process.Recent studies have shed light on the intricate regulatory network governing the assembly and activity of the exocyst complex across various fungal species.

Post-translational modifications, such as phosphorylation, ubiquitination and lipid modifications, have emerged as key regulators of exocyst complex function. Phosphorylation, in particular, has been shown to modulate the stability, localization and activity of exocyst subunits in fungi [51,86]. Recent studies have elucidated the roles of specific protein kinases, including cyclin-dependent kinase (CDK) family members, protein kinase A (PKA), and mitogen-activated protein kinase (MAPK) cascades, in mediating the phosphorylation of exocyst subunits across fungal species [39,99,100,101,102,103]. In *S. cerevisiae*, phosphorylation of the Exo84 subunit by Cdk1 kinase regulates the assembly and disassembly of the exocyst complex during the cell cycle [22,104]. The association of the exocyst complex with the plasma membrane or secretory vesicles can influence its assembly and disassembly. In both budding and fission yeast, the exocyst subunits Sec3 and Exo70 have been shown to interact with phospholipids, which is important for their localization and regulation of the exocyst complex [51,71,72,105]. A recent study on the fission yeast *Schizosaccharomyces pombe* has demonstrated that Orb6 phosphorylation of Sec3 contributes to exocyst function in coordination with the exocyst protein Exo70. This suggests that Orb6 promotes polarized growth by regulating membrane trafficking at multiple levels [106].

Moreover, regulatory factors such as small GTPases, kinases and phosphatases play crucial roles in modulating exocyst assembly and activity. Recent research has highlighted the involvement of small GTPases like Rho3 in regulating exocyst localization and function in yeast, as well as the impact of kinases and phosphatases on exocyst subunit phosphorylation and dephosphorylation dynamics [70,107,108]. In *S. cerevisiae*, the small GTPases Sec4 and Rho1 have been shown to interact with the exocyst subunits and regulate its assembly and localization [80,109]. In *C. albicans*, the small GTPase Rho1 interacts with the exocyst subunit Sec3 and is involved in regulating exocyst assembly and localization [80,110]. In *A. nidulans*, the small GTPase RacA regulates the localization and dynamics of the exocyst complex, which is important for polarized growth and secretion [111,112]. Furthermore, protein-protein interactions within the exocyst complex and with regulatory proteins are integral for its proper function. Recent studies have unveiled novel interactions between exocyst subunits and polarity-establishment components, shedding light on their roles in cellular polarity regulation [51]. The binding of other regulatory proteins to the exocyst subunits can modulate the assembly and disassembly of the complex. For example, in *S. cerevisiae*, the Sec3 subunit interacts with the polarisome complex, which is involved in regulating exocyst dynamics [113]. Additionally, insights into the stability-regulating interactions within the exocyst complex, such as Sec6–Sec8 and Sec10–Sec15 interactions, have provided deeper understanding of exocytosis and polarized growth mechanisms [70,74,75,114]. Moreover, signaling pathways, including the TOR pathway, cell cycle machinery, cAMP-PKA pathway, MAPK cascade, and lipid signaling pathways, exert regulatory control over exocyst assembly and activity. Recent research has elucidated the intricate crosstalk between these signaling pathways and the exocyst complex, revealing their roles in modulating exocyst function in response to environmental cues and cellular stresses [5,115,116].

## 5. Roles of Fungal Exocyst Complex in Cellular Secretion

**Figure 1:**
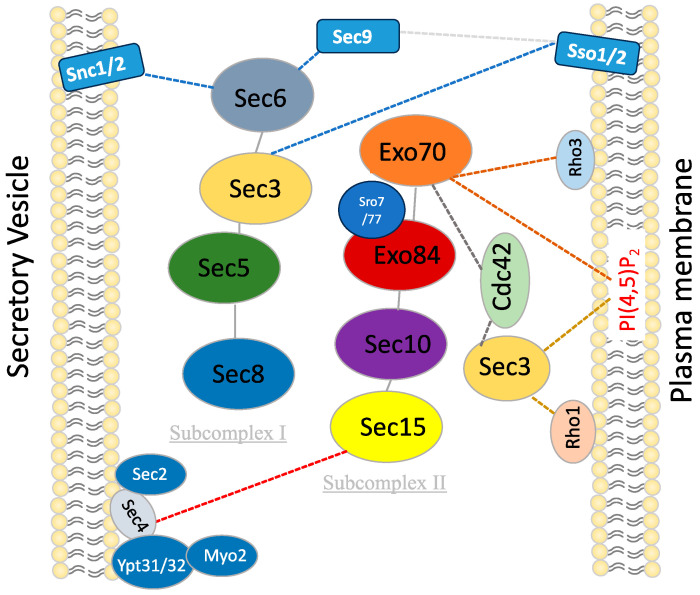
Interaction of exocyst complex with secretory vesicles and cytoplasmic membranes in yeast.

**Figure 2:**
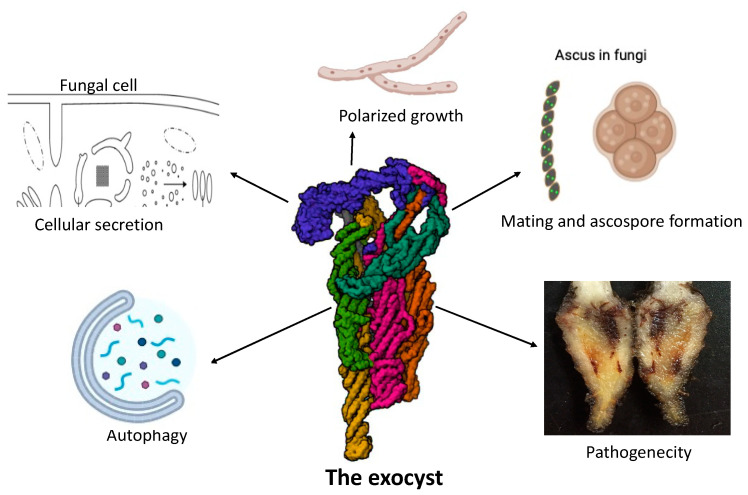
Various functions of the exocyst in fungi.

In fungi, the exocyst complex plays a pivotal role in cellular secretion processes, impacting growth, morphogenesis and pathogenicity. This complex ensures the spatial and temporal coordination of vesicle fusion with the plasma membrane. Recent studies have highlighted the exocyst’s involvement in the regulation of polarized growth, particularly in filamentous fungi where it directs vesicle trafficking to the growing hyphal tips [17,117]. Additionally, the exocyst is implicated in cell wall integrity maintenance and response to environmental stress, crucial for fungal survival and adaptation [31] (see Figure 1 and Figure 2).

### 5.1. Exocyst Complex Promotes SNARE-Mediated Membrane Fusion

The complete assembly of the exocyst complex is accompanied by vesicle tethering to the plasma membrane, with subsequent membrane fusion mediated by SNARE proteins both on the vesicle and plasma membrane [118,119,120,121]. SNARE proteins are classified based on their localization: v-SNAREs on vesicles and t-SNAREs on target membranes. In yeast, the t-SNARE proteins Sso1/2 (homologous to Syntaxin in mammals) and Sec9 (homologous to SNAP-25 in mammals) mediate vesicle-plasma membrane fusion. The v-SNARE proteins involved in this fusion include Snc1/2 (homologous to VAMP/Synaptobrevin in mammals) [122,123]. During vesicle-plasma membrane fusion, Sso forms a binary t-SNARE complex with Sec9, which then binds to Snc to assemble the complete SNARE complex. Besides bringing v-SNARE and t-SNARE proteins into spatial proximity, the exocyst facilitates SNARE complex assembly and membrane fusion through its interactions with SNAREs and SNARE-regulated proteins. The exocyst subunit Sec3 interacts with Sso2 via its PH domain, inducing a conformational change in Sso2 that promotes the formation of the binary complex with Sec9, thus facilitating membrane fusion [61]. Sec6 binds directly to Sec9, the Sso1-Sec9 binary complex, the Sec9-Sso1-Snc2 ternary complex and the SM (Sec1/Munc-18) protein Sec1, which regulates SNARE assembly [59,124]. However, there are currently no conclusive experimental data in fungi supporting the exocyst’s role in SNARE assembly and SNARE-mediated membrane fusion.

### 5.2. Exocyst-Mediated Vesicle Trafficking in Fungi

Generally, in fungi, exocytosis is essential for growth, development, nutrient acquisition, response to environmental cues and interactions with other organisms, including pathogenesis in the case of fungal pathogens. Fungal exocytosis is mediated by the exocyst complex and involves precise spatial and temporal regulation to ensure efficient secretion and proper cellular function. Deletion or mutation of any exocyst subunit leads to defective cytokinesis and accumulation of secretory vesicles, highlighting its role in vesicle transport, tethering and membrane fusion [65]. Genetic and biochemical studies in fungi indicate that the exocyst complex travels with secretory vesicles to specific sites on the plasma membrane where exocytosis occurs [37,105,125]. In fungi, the exocyst is located at the hyphal tip and subapically, playing a crucial role in cellular expansion and facilitating the secretion of hydrolytic enzymes from septa [14,17,94].

The exocyst complex is crucial for vesicle targeting and tethering through specific interactions with secretory vesicles and the plasma membrane [114]. Live-cell imaging has shown that Sec8, a component of the exocyst, is transported with secretory vesicles to the plasma membrane and remains there until completion of membrane fusion, suggesting its role in vesicle tethering and fusion [126]. Additionally, studies in yeast demonstrated that ectopic targeting of Sec3 to mitochondria or peroxisomes recruits secretory vesicles to these organelles, supporting the exocyst’s role in vesicle targeting [78]. In a recent study, Rossi et al. (2020) reconstituted an in vitro vesicle tethering assay using purified Sro7, and exocyst proteins with fluorescently labeled post-Golgi vesicles from yeast [127]. This assay highlighted the necessity of functional Sec4 and the R-SNAREs Snc1/2 for exocyst-mediated tethering. Further studies have shown that the exocyst-mediated tethering in yeast involves both Rab GTPase and SNARE proteins, which originate from the same membrane. Additionally, a Sac1-sensitive phosphoinositide, likely PI4P, plays a crucial role on the opposing membrane. This suggests a specific model for exocyst orientation and its points of contact between membranes during vesicle tethering [128]. Another study by Zhang et al. (2005) indicates that the exocyst complex and Sro7 function in parallel and directly interact via the Exo84 subunit of the exocyst. Sro7, although not stably associated with the exocyst, transiently interacts with Exo84, facilitating vesicle tethering to the target membrane [125]. Sro7 and Rho GTPases, both located at sites of polarized growth, enhance exocyst tethering by strengthening its binding to Sec4 and Snc1/2 on the vesicle surface [60]. Both exocyst and Sro7 facilitate SNARE-mediated fusion by promoting the assembly of SNARE monomers into fusion complexes at these growth sites, following the initial docking event [114,129]. Interactions between exocyst subunits and phospholipids or small G proteins on vesicle and plasma membranes provide a physical basis for vesicle tethering [47,72,130].

Two key subunits of the exocyst, Sec3 and Exo70, directly bind to PI(4,5)P2 (Phosphatidylinositol 4,5-bisphosphate), a phospholipid located on the inner leaflet of the plasma membrane [51]. Sec3 interacts with PI(4,5)P2 through an evolutionarily conserved PH-domain-like region at its N-terminus, while Exo70 interacts with PI(4,5)P2 through a patch of basic residues at its C-terminus [47,51,71,130]. This interaction is crucial for anchoring the exocyst complex to specific sites on the membrane where exocytosis will occur. Once the Sec3 and Exo70 are in place, the assembly of the remaining exocyst components proceeds as secretory vesicles arrive at these predetermined sites. Sec3 and Exo70 also interact with Rho GTPases, localized to the plasma membrane [45]. These two interactions of the exocyst with phospholipids and small GTPases are essential for cell growth and secretion, as they facilitate proper targeting and fusion of secretory vesicles with the plasma membrane [72]. While Sec3 and some of the Exo70 proteins associate with the plasma membrane via PI(4,5)P2, other members of the exocyst associate with the secretory vesicles and require myosin V-mediated transport along actin cables for their localization to sites of growth [45]. Domain analysis has shown that budding yeast requires at least one domain from either Sec3 or Exo70 that is capable of binding Cdc42 and PIP2 for viability, highlighting the critical role of these two proteins as spatial landmarks for polarized exocytosis [72]. Conversely, disruption of the interaction between Sec3 and Exo70 with PIP2 or a reduction in intracellular PIP2 content impedes the assembly of the exocyst on the plasma membrane, resulting in the dissociation of the exocyst from the plasma membrane thereby impeding secretion [51].

On the vesicle, the exocyst interacts with the Rab GTPase Sec4 via the Sec15 subunit [70,131] and with the v-SNAREs Snc1/2 via the Sec6 subunit [115]. Sec6 binds SNARE proteins and promotes SNARE complex assembly [124] and also binds the SNARE interactor Sec1 [59]. Theses interactions ensure that the exocyst associates only with vesicles carrying both key ligands [115]. For tethering vesicles to the target membrane, the exocyst binds to proteins and lipids on the plasma membrane, including the phospholipid PI(4,5)P2 and the Rho GTPases Rho3, Cdc42 and Rho1, through Exo70 and Sec3 subunits [51,72,81]. Additionally, the exocyst interacts with the target SNAREs Sec9 and Sso1 through the Sec6 and Sec3 subunits, respectively [61,124] (see Table 2 & Figure 1).

In filamentous fungi, polarized secretion is crucial for fungal apical growth, involving the directed movement of secretory vesicles to the vesicle supply center (VSC), a densely populated region of vesicles located at the hyphal tip [7,15,132]. The Spitzenkörper, acting as a VSC, orchestrates the trafficking of secretory vesicles toward the hyphal tip for cell wall biogenesis [110]. A prominent example of this intricate process is observed in the filamentous fungus *N. crassa*, where chitosomes accumulate beneath the hyphal tip within a specialized structure called the Spitzenkörper [7,132]. The Spitzenkörper serves as a vesicle-sorting center, orchestrating the directional growth of hyphae by regulating the delivery of secretory vesicles containing essential cell wall biosynthesis machinery. This dynamic structure directs polarized growth by concentrating chitosomes and other secretory organelles beneath the hyphal tip, ensuring localized deposition of cell wall components for elongation [7,132]. Interestingly, in the pathogenic yeast *C. albicans*, the Spitzenkörper represents a distinct entity from the exocyst and polarisome, two other molecular complexes involved in polarized growth regulation [110]. This distinct localization highlights the diversity of mechanisms employed by fungi to achieve polarized growth and underscores the complexity of cellular organization in response to environmental cues. Further studies in various fungal species, including *C. albicans*, *A. nidulans* and *F. odoratissimum*, provided insights into the role of the exocyst complex in vesicle trafficking and cellular secretion [17,133]. In *C. albicans*, the exocyst complex is essential for hyphal morphogenesis, biofilm formation and secretion of virulence factors [43,116]. A study shows that *C. albicans* Sec15 interacts directly with the polarity determinant Bem1 and the type V myosin Myo2. The researchers found that disrupting this interaction by shutting off *SEC15* expression results in the mislocalization of Bem1-GFP. These findings highlight the important role of Sec15 in polarized cell growth by establishing a direct functional link between bud-site selection and exocytosis [43]. Similarly, in *A. nidulans* and *F. odoratissimum*, the exocyst complex coordinates the secretion of hydrolytic enzymes and secondary metabolites, crucial for fungal growth and pathogenesis [17,134] (see Table 2).

**Table 2:** Interacting partners of the exocyst subunits in fungi.

Exocyst Subunit	Interactor	Cellular Process	References
Sec3	Rho1, Cdc42, Sso1/2, PI(4,5)P2	Facilitates plasma membrane binding and SNARE assembly	[61,72,127]
Sec5	N/A *	Mediates the interaction of the two subcomplexes of exocyst	[23,135]
Sec6	Snc1/2, Sec1, Sec9	Facilitates SNARE assembly.	[59,124,136]
Sec8	Rho3, Rho4	Promote glucanase secretion to the proper area during a specific time in the cell cycle.	[137,138]
Sec10	N/A *	N/A *	
Sec15	Ypt31/32, Sec2, Sec4, Myo2 and Bem1		[43,44,70,139,140]
Exo70	Rho3, Cdc42, PI(4,5)P2		[81,124,141]
Exo84	Sro7/Sro77		[125,127,135]

* N/A: NOT applicable or NOT available.

The schematic outlines the pivotal interactions and cellular processes involving subunits of the exocyst complex in yeast. Each subunit engages specific interactors and contributes to distinct cellular functions. Sec3 interacts with Rho1, Cdc42, Sso1/2, and PI(4,5)P2, facilitating plasma membrane binding and SNARE assembly. Sec5 mediates interaction between the two subcomplexes of Exocyst. Sec6 interacts with Snc1/2, Sec1, and Sec9 to facilitate SNARE assembly. Sec8 interacts with Rho3/Rho4, promoting glucanase secretion to the appropriate location during specific cell cycle phases. Sec15 interacts with Ypt31/32, Sec2, Sec4, Myo2, and Bem1, guiding the Exocyst to secretory vesicles and playing a central role in polarized growth and exocytosis. Exo70 interacts with Rho3, Cdc42, and PI(4,5)P2 to facilitate initial plasma membrane binding. Exo84 interacts with Sro7/Sro77 to facilitate SNARE assembly.The exocyst subunits are shown in different colors. the dotted lines indicate the reported interactions.

### 5.3. Cargos of the Exocyst Complex

The exocyst complex serves as a crucial transporter of diverse cargo molecules, encompassing proteins, lipids and carbohydrates, to the plasma membrane in fungi [45,49,77]. These cargo molecules play multifaceted roles in fungal biology, from cell wall synthesis to the secretion of virulence factors during pathogenesis. Among the well-studied cargo molecules is chitin synthase 3 (Chs3), an enzyme pivotal in cell wall biosynthesis. The exocyst complex facilitates the delivery of Chs3 to the plasma membrane where it catalyzes the synthesis of chitin, a key component of fungal cell walls. This process is indispensable for maintaining cell wall integrity and rigidity and is vital for fungal growth and morphogenesis [141]. Moreover, the exocyst complex transports various secreted proteins implicated in fungal pathogenesis. In pathogenic fungi like *C. albicans*, the exocyst mediates the secretion of adhesins and invasions, facilitating the fungal cell attachment to host tissues and invasion of host cells. Precise targeting of these virulence factors to the plasma membrane is crucial for establishing fungal infections and evading host immune responses [140,142,143]. Additionally, the exocyst complex is involved in the secretion of extracellular enzymes crucial for nutrient acquisition and environmental adaptation in fungi [28,51,109]. In filamentous fungi, the exocyst complex is involved in the secretion of hydrolytic enzymes, including proteases and lipases, which are essential for degrading complex substrates into simpler molecules that can be absorbed by fungal cells [5,28]. For instance, in species like *A. nidulans* and *F. odoratissimum*, the exocyst complex mediates the secretion of hydrolytic enzymes critical for nutrient acquisition and environmental adaptation [17,92]. Recent studies shed light on the regulatory mechanisms governing the secretion of hydrolytic enzymes by the exocyst complex [108,144]. For example, in *A. oryzae*, an exocyst subunit, Sec3, has been implicated in the secretion of α-amylase at the septum [14]. This finding underscores the diverse roles of the exocyst complex in orchestrating the secretion of hydrolytic enzymes in filamentous fungi.

Furthermore, the exocyst functions at multiple stages of cytokinesis. Through phosphorylation of Exo84, Cdk1 regulates the assembly of the exocyst complex, halting exocytosis before the transition from metaphase to anaphase [100]. At telophase, the exocyst directs vesicle trafficking to the cell–cell junction for abscission [137,145,146]. These insights deepen our understanding of the cargo molecules transported by the exocyst complex and their pivotal roles in fungal biology. Further exploration of these cargo molecules and their interactions with the exocyst complex holds promise for elucidating the molecular mechanisms underlying fungal physiology and pathogenesis.

## 6. The Role of the Exocyst Complex in Polarized Growth

Fungi comprise a variety of organisms known for their remarkable adaptability to different ecological niches, mainly due to their capacity for polarized growth. This dynamic process, which is essential for hyphal elongation, branching and morphogenesis, allows fungi to thrive in different habitats and connect with their environment. In most filamentous fungi, hyphal growth is organized around a specialized organelle called the Spitzenkörper (SPK), a focal point of vesicle trafficking and secretion [7,9]. The SPK consists of Golgi-derived vesicles containing enzymes, lipids and polysaccharides for membrane and cell wall synthesis and plays a central role in fungal morphogenesis [147]. Recent studies highlighted the importance of the exocyst in facilitating the secretion of enzymes and other proteins necessary for fungal growth and pathogenicity. For instance, in *F. odoratissimum*, a notorious pathogen causing banana wilt, all the eight exocyst components have been found to localize not only at the tips ahead of the Spitzenkörper in growing hyphae but also at the outer edges of septa in mature hyphae. This dual localization suggests a complex role in both active secretion during hyphal growth and maintenance processes in mature cells [17]. In *N. crassa* and *A. nidulans*, the exocyst localizes to the growing tips of hyphae, where it directs the delivery of cell wall materials and secretory vesicles, facilitating hyphal extension and branching [15,92]. Furthermore, the exocyst’s role in fungal polarized growth extends beyond hyphal morphogenesis to various developmental processes and environmental responses. In pathogenic fungi, such as *C. albicans* and *Cryptococcus neoformans*, the exocyst contributes to virulence-related processes, including invasive growth, biofilm formation and host colonization [148,149]. Additionally, in symbiotic fungi, such as arbuscular mycorrhizal fungi, the exocyst is involved in establishing symbiotic interfaces with plant roots, facilitating nutrient exchange and mutualistic interactions [150,151]. Moreover, the exocyst’s dynamic regulation in response to environmental cues and physiological conditions underscores its versatility and adaptability in fungal polarized growth. In response to nutrient availability, stress signals and developmental cues, the exocyst complex undergoes spatial reorganization and modulates its activity to regulate polarized growth patterns and adapt to changing conditions [152,153,154]. Recent studies have highlighted the importance of polarized growth in fungal biology and pathogenicity. For instance, in filamentous fungi such as *Aspergillus fumigatus*, polarized growth at the hyphal tip is crucial for hyphal elongation and invasion of host tissues during infection [155]. Similarly, in plant-pathogenic fungi like *M. oryzae*, polarized growth facilitates the formation of specialized infection structures called appressoria, which exert mechanical force to breach the host surface and initiate colonization [156] (see Figure 2).

## 7. Role of the Exocyst Complex in Autophagy in Fungi

In addition to its role in exocytosis (the process by which cells export molecules), the exocyst complex plays a crucial role in autophagy (the process of degrading and recycling cellular components) in fungi. The exocyst complex contributes to the regulation and facilitation of this cellular process. Autophagy involves the formation of autophagosomes, which engulf cellular components and deliver them to the vacuole for degradation and recycling. The involvement of the exocyst in autophagy includes several key aspects. Firstly, the exocyst is essential for the efficient formation and maturation of autophagosomes. It facilitates the trafficking and tethering of vesicles required for autophagosome assembly, involving the interaction of the exocyst subunits with some key autophagy-related proteins [157]. Secondly, the exocyst mediates the transport of vesicles containing autophagic machinery components to the pre-autophagosomal structure (PAS), a site where autophagosomes are nucleated, which is crucial for the proper assembly of autophagosomes [158]. Additionally, by interacting with SNARE proteins, the exocyst complex assists in the membrane fusion events necessary for autophagosome completion, ensuring that autophagosomes fuse with vacuoles to deliver cargos for degradation [125]. The exocyst also interacts with various autophagy-related (Atg) proteins like Atg9 which is involved in membrane delivery to the PAS, and these interactions are critical for coordinating the dynamics of membrane addition during autophagosome formation [159]. Studies have demonstrated that exocyst components are localized to the PAS and are required for the proper formation of autophagosomes. For example, the exocyst subunit Sec3 interacts with Atg17, an essential protein in autophagy initiation [160]. Moreover, the exocyst is involved in tethering autophagic vesicles to specific sites on the vacuole membrane, facilitating their fusion, which is crucial for the final stages of autophagy where autophagosomes fuse with vacuoles to deliver their contents [161]. A recent study on autophagosome biogenesis in yeast exocyst mutants suggests the involvement of an exocyst subcomplex composed of all exocyst subunits except Sec15, Exo70 and Exo84 in yeast autophagy [162]. The molecular mechanisms underlying the exocyst’s role in autophagy are not only complex but may also vary depending on the specific cell or tissue type. The exocyst complex’s involvement in both exocytosis and autophagy underscores its versatility in managing membrane dynamics and vesicular trafficking. While traditionally associated with exocytosis, the complex’s role in autophagy highlights its broader function in cellular homeostasis and stress response. By facilitating membrane trafficking and fusion events, the exocyst complex ensures the smooth operation of both secretion and degradation pathways. Understanding the exocyst complex’s role in autophagy is still evolving, with ongoing research providing deeper insights (see Figure 2).

## 8. Roles of the Exocyst Complex in Mating and Ascospore Formation

The exocyst complex plays pivotal roles in various cellular processes, particularly in vesicle trafficking and membrane fusion. Its functions extend to crucial developmental stages in fungi, such as mating and ascospore formation. In yeast, particularly the fission yeast *S. pombe*, exocyst components have been reported to be integral to these processes. Studies indicate that mutants of these components exhibit reduced mating efficiency and abnormal or absent ascospore formation, highlighting the essential nature of the exocyst in these reproductive events [163]. Furthermore, the exocyst is vital for the development of the forespore membrane (FSM), a precursor to the ascospore membrane, underscoring its importance in cellular differentiation during reproduction [163,164]. Similarly, in the plant pathogenic fungus *Bipolaris maydis*, the exocyst complex, particularly the BmSec5 component, appears to play a comparable role in ascospore delimitation and elongation. This suggests a conserved function of the exocyst across different fungal species, despite some variations in mating behavior and ascospore development between yeast and *B. maydis* [63] (see Figure 2).

## 9. The Role of the Exocyst Complex in Fungal Pathogenicity

The role of the exocyst in fungal pathogenicity represents a critical area of study in understanding how fungal pathogens interact with their hosts and cause disease. Fungi have evolved sophisticated mechanisms to infect various hosts, including plants, animals and humans, posing significant threats to global health, agriculture and ecosystems [39]. The ability of fungal pathogens to colonize host tissues, evade immune defenses and cause disease is intricately linked to their secretion systems, including the exocyst complex [11,28]. In pathogenic fungi, the exocyst assumes specialized roles that significantly impact the fungal ability to infect and cause disease. Even though *S. cerevisiae* lacks pathogenicity, insights into its exocyst functions provide valuable understandings applicable to more complex fungi. In *S. cerevisiae*, the exocyst is indispensable for maintaining cell polarity, cytokinesis and overall cell viability, with Sec3 standing out as a notable exception [11,28,33]. In contrast to *S. cerevisiae*, *Schizosaccharomyces pombe* exhibits species-specific differences in exocyst component requirements, with exo70 being non-essential. Here, the exocyst is crucial for directing vesicles to specific plasma membrane sites, thus facilitating cell growth and division [144,146,165]. In *C. albicans*, the exocyst complex assumes a critical role in hyphal growth, a significant virulence factor allowing the fungus to invade host tissues and evade immune responses. Disruption of exocyst components, such as Sec3, significantly impairs hyphal growth and reduces pathogenicity, emphasizing the exocyst’s importance in morphogenesis and pathogenicity [144]. Similarly, the exocyst in *Aspergillus niger* is involved in polar growth and secretion, with Sec3 being non-essential, indicating potential functional redundancy or alternative pathways. The exocyst’s role in mediating enzyme secretion is crucial for pathogenic fungal ability to degrade host tissues and facilitate infection [15,166,167]. In *N. crassa*, the exocyst complex is indispensable for polarized growth and asexual spore formation. Notably, Sec5 is non-essential in *N. crassa*, suggesting variations in exocyst dependency among fungi. The exocyst directs vesicles to hyphal tips, ensuring proper growth and development [16,146]. Moreover, in *M. oryzae*, the exocyst is critical for appressorium formation, a specialized structure facilitating host tissue penetration. Both exo70 and sec5 are non-essential in *M. oryzae*, hinting at alternative mechanisms for vesicle trafficking. Insights into the exocyst’s role provide valuable understanding of *M. oryzae* pathogenic strategies and potential disease control targets [15,166,167]. In *F. odoratissimum*, the exocyst complex is believed to fulfill functions similar to those observed in other fungi, contributing to polarized growth and the secretion of virulence factors crucial for host infection and colonization [17]. Deletion of Exo70 results in a significant decrease in the secretion and activity of various enzymes, including endoglucosidase and amylase, across different organisms, while deletion of sec5 only minimally impacts amylase activity [17]. These findings underscore the critical involvement of the exocyst complex in regulating cellular mechanisms responsible for enzyme production and secretion. Further investigations in *Botrytis cinerea* have also revealed that exocyst complex is pivotal for secreting enzymes that degrade plant cell walls, facilitating tissue maceration and nutrient acquisition [36,63]. Disruption of Exo70 in *B. cinerea* significantly impairs pathogenicity, highlighting the complex’s vital role in the fungal life cycle and disease progression. Additionally, deletion of SEC5 in *B. cinerea* results in a notable reduction in hyphal growth and a slight decrease in pathogenicity [63].

Fungi employ a variety of virulence factors to establish infection within host tissues, evade immune surveillance and acquire nutrients. The exocyst complex regulates the secretion of virulence-associated molecules such as hydrolytic enzymes, toxins and adhesion proteins, which are essential for host tissue invasion and establishment of infection [168]. In the dynamic interplay between fungi and their hosts, the exocyst complex is implicated in numerous processes that are essential for both mutualistic symbioses and pathogenic interactions [166,169]. For instance, in mutualistic symbioses such as mycorrhizal associations, the exocyst is involved in the establishment and maintenance of symbiotic interfaces, facilitating nutrient exchange between plants and mycorrhizal fungi [150,151]. Studies in model legume-rhizobia and arbuscular mycorrhizal symbioses have highlighted the importance of exocyst-mediated vesicle trafficking in the formation of symbiotic structures and the regulation of symbiotic signaling pathways [150,151]. Conversely, in pathogenic interactions, the exocyst complex plays critical roles in fungal colonization, host invasion and disease development. Pathogenic fungi deploy an array of virulence factors to breach plant defenses and establish infection. Recent studies have implicated the exocyst in the secretion of effector proteins, cell wall-degrading enzymes, and toxins involved in plant pathogenesis [46,170]. For example, in the rice blast fungus *M. oryzae*, the exocyst subunit Exo70 is required for the secretion of effectors involved in host colonization and virulence [16,46,166]. Moreover, the exocyst complex is involved in the regulation of plant immune responses to fungal pathogens. Plant cells recognize conserved microbial molecules, known as pathogen-associated molecular patterns (PAMPs), triggering immune responses to restrict pathogen proliferation. The exocyst-mediated secretion of defense-related molecules, including antimicrobial peptides, reactive oxygen species and defense hormones, modulates plant immunity and contributes to the outcome of plant-fungal interactions [171,172].

The exocyst complex also plays a critical role in the pathogenesis of *C. albicans* and *Aspergillus fumigatus* during interactions with their hosts. *C. albicans* is a major opportunistic fungal pathogen in humans that causes infections ranging from superficial mucosal infections to life-threatening systemic diseases, especially in immunocompromised individuals [173]. *Aspergillus fumigatus*, on the other hand, is a ubiquitous mold responsible for a variety of respiratory infections, particularly in immunocompromised patients [174]. In both fungi, the exocyst complex is essential for virulence and adaptation to host environments. It facilitates the secretion of virulence factors, adherence to host cells and evasion of host immune responses [175]. Specifically, in *C. albicans*, the exocyst complex is involved in the secretion of hypha-specific proteins that are critical for tissue invasion and biofilm formation, enhancing the ability of the fungus to colonize and persist within host tissues [116,176]. In *Aspergillus fumigatus*, the exocyst complex is similarly implicated in the secretion of allergens and toxins that contribute to host colonization and disease progression. Studies have shown that the complex coordinates the assembly and secretion of hydrolytic enzymes and immunomodulatory factors, enabling the fungus to breach host barriers and modulate immune responses [1,177] (see Figure 2).

The exocyst, an octameric complex consisting of eight subunits, has diverse roles in fungi, including protein trafficking, vesicle tethering to the plasma membrane for secretion, polarized growth, mating and ascospore formation, autophagy and fungal pathogenesis.This detailed representation of the exocyst’s multifunctionality underscores its critical role in fungal biology, influencing both normal growth processes and pathogenic mechanisms.

## 10. Prevailing Challenges in Understanding the Exocyst Complex

Despite its significance, understanding the exocyst complex poses several challenges [12]. Firstly, its structural complexity, composed of eight subunits (Sec3, Sec5, Sec6, Sec8, Sec10, Sec15, Exo70, and Exo84), makes it difficult to determine the precise structure and interactions at a molecular level due to their size and the dynamic nature of their interactions [51]. Secondly, the exocyst complex undergoes various conformational changes during the vesicle tethering process, making it challenging to capture these dynamic interactions in real-time and understand their regulation [6]. Additionally, the localization and timing of exocyst assembly and function are tightly regulated within the cell, necessitating advanced imaging and molecular techniques to understand these spatial and temporal aspects [8].Functional redundancy and compensation within cells often obscure the specific roles of the exocyst complex, requiring sophisticated genetic and biochemical approaches to dissect these overlapping functions [178]. Furthermore, the exocyst complex might have different roles and regulatory mechanisms in different cell types, demanding diverse model systems and experimental conditions to study these variations comprehensively [55]. Post-translational modifications (PTMs) such as phosphorylation, ubiquitination, and glycosylation add another layer of complexity, as understanding how these PTMs affect the function and regulation of the exocyst is an ongoing challenge [179]. The exocyst complex is also implicated in various diseases, including cancer and neurological disorders, necessitating a bridge between basic molecular studies and clinical research to understand its role in these pathological conditions [109]. Lastly, current technological limitations in high-resolution imaging, structural biology, and molecular biology techniques hinder the detailed study of the exocyst complex, emphasizing the need for advances in cryo-electron microscopy, super-resolution microscopy, and other cutting-edge technologies to overcome these challenges [141]. Addressing these challenges requires a multidisciplinary approach, combining structural biology, cell biology, genetics, biochemistry, and advanced imaging techniques to unravel the complexities of the exocyst complex and its role in cellular processes [109].

## 11. Conclusions

The exocyst complex in fungi plays a pivotal role in cellular secretion, growth and pathogenesis, offering intriguing possibilities and challenges for research in fungal biology and biotechnology. Understanding its evolutionary conservation across fungal species through comparative genomics and phylogenetic studies reveals insights into its fundamental functions and adaptations [70]. This evolutionary perspective underscores its significance in diverse cellular processes and its potential as a target for novel antifungal strategies. Structurally, integrative modeling techniques have provided unprecedented insights into the exocyst complex’s molecular architecture and functional dynamics. Combining data from various sources, including electron microscope density maps, chemical cross-links and computational models, researchers have elucidated the spatial organization and interactions of exocyst subunits [24]. These advances are crucial for understanding how the exocyst coordinates vesicle trafficking and membrane fusion at precise cellular locations essential for fungal growth and morphogenesis. Regulation of the exocyst complex involves intricate networks of signaling pathways and regulatory factors that vary among fungal species. Post-translational modifications like phosphorylation and ubiquitination dynamically modulate exocyst function in response to environmental cues and developmental signals [42]. Additionally, transcriptional regulation of exocyst components by transcription factors further contributes to its regulatory complexity [28].

These regulatory mechanisms not only govern fungal growth and development but also influence pathogenicity, making them attractive targets for therapeutic interventions. Biotechnologically, exploiting exocyst-mediated secretion pathways holds promise for enhancing protein production, optimizing secretion processes and designing innovative drug delivery systems [135]. Understanding the exocyst functions in fungal pathogenesis is particularly relevant for developing targeted therapies against fungal infections. By disrupting specific exocyst components or regulatory pathways, researchers can potentially mitigate fungal virulence while minimizing host cell damage. Despite significant advancements, challenges persist in exocyst research, including the understanding the functional redundancy and specificity of the exocyst subunits and unraveling unconventional secretion pathways. Addressing these challenges will require integrating advanced imaging techniques such as cryo-electron microscopy and super-resolution microscopy with biochemical and biophysical approaches [135]. These interdisciplinary efforts aim to uncover the full spectrum of exocyst-mediated processes and pave the way for transformative discoveries in fungal biology and biotechnology.

Currently, there is ongoing debate about the precise mechanism of exocyst complex-mediated vesicle tethering, with species-specific regulatory mechanisms requiring further investigation. The use and improvement of innovative technologies and methodologies, such as gene editing techniques, super-resolution microscopy imaging, in vitro tethering reconstruction, and single-molecule dynamic analysis, are expected to provide insights into these unresolved questions.

In conclusion, the exocyst complex represents a cornerstone of fungal biology with profound implications for medicine and biotechnology. Continued exploration of its evolutionary conservation, structural organization, regulatory mechanisms and biotechnological potential will advance our understanding of fungal pathogenesis and drive innovations in antifungal therapies and biotechnological applications. Interdisciplinary collaborations and innovative research strategies will be crucial for overcoming current challenges and harnessing the exocyst complex’s full potential in fungal research and biotechnology.

## Figures and Tables

**Table 1:** Overview of the core components of the exocyst complex and their functions in fungi.

Exocyst Subunit	Function in Fungi	References
Sec3	Marks the site of exocytosis and recruits the exocyst complex	[5,61,62]
Sec5	Involved in vesicle tethering and fusion	[11,63]
Sec6	Interacts with the SNARE complex to facilitate membrane fusion	[64,65]
Sec8	Stabilizes the exocyst complex and links it to the actin cytoskeleton	[11,28]
Sec10	Involved in vesicle tethering and fusion	[66,67]
Sec15	Links the exocyst to the Rab GTPase that marks the vesicle	[44,68]
Exo70	Interacts with the plasma membrane and the actin cytoskeleton	[69]
Exo84	Interacts with the Rab GTPase and helps tether the vesicle to the exocyst	[5,70]

## References

[1] Liebana-Jordan M., Brotons B., Falcon-Perez J.M., Gonzalez E. (2021). Extracellular Vesicles in the Fungi Kingdom. Int. J. Mol. Sci..

[2] Lai Y., Jiang B., Hou F., Huang X., Ling B., Lu H., Zhong T., Huang J. (2023). The emerging role of extracellular vesicles in fungi: A double-edged sword. Front. Microbiol..

[3] Abubakar Y.S., Sadiq I.Z., Aarti A., Wang Z., Zheng W. (2023). Interplay of transport vesicles during plant-fungal pathogen interaction. Stress Biol..

[4] Novick P., Field C., Schekman R. (1980). Identification of 23 complementation groups required for post-translational events in the yeast secretory pathway. Cell.

[5] Heider M.R., Munson M. (2012). Exorcising the Exocyst Complex. Traffic.

[6] Wu B., Guo W. (2015). The Exocyst at a Glance. J. Cell Sci..

[7] Riquelme M., Aguirre J., Bartnicki-García S., Braus G.H., Feldbrügge M., Fleig U., Hansberg W., Herrera-Estrella A., Kämper J., Kück U. (2018). Fungal Morphogenesis, from the Polarized Growth of Hyphae to Complex Reproduction and Infection Structures. Microbiol. Mol. Biol. Rev..

[8] Liu D., Li X., Shen D., Novick P. (2018). Two subunits of the exocyst, Sec3p and Exo70p, can function exclusively on the plasma membrane. Mol. Biol. Cell..

[9] Takeshita N. (2016). Coordinated process of polarized growth in filamentous fungi. Biosci. Biotechnol. Biochem..

[10] Chen J., Yamagata A., Kubota K., Sato Y., Goto-Ito S., Fukai S. (2017). Crystal structure of Sec10, a subunit of the exocyst complex. Sci. Rep..

[11] TerBush D.R., Maurice T., Roth D., Novick P. (1996). The Exocyst is a multiprotein complex required for exocytosis in Saccharomyces cerevisiae. EMBO J..

[12] He B., Guo W. (2009). The exocyst complex in polarized exocytosis. Curr. Opin. Cell Biol..

[13] Köhli M., Galati V., Boudier K., Roberson R.W., Philippsen P. (2008). Growth-speed-correlated localization of exocyst and polarisome components in growth zones of *Ashbya gossypii* hyphal tips. J. Cell Sci..

[14] Hayakawa Y., Ishikawa E., Shoji J., Nakano H., Kitamoto K. (2011). Septum-directed secretion in the filamentous fungus *Aspergillus oryzae*. Mol. Microbiol..

[15] Riquelme M., Bredeweg E.L., Callejas-Negrete O., Roberson R.W., Ludwig S., Beltrán-Aguilar A., Seiler S., Novick P., Freitag M. (2014). The *Neurospora crassa* exocyst complex tethers Spitzenkörper vesicles to the apical plasma membrane during polarized growth. Mol. Biol. Cell.

[16] Gupta Y.K., Dagdas Y.F., Martinez-Rocha A.L., Kershaw M.J., Littlejohn G.R., Ryder L.S., Sklenar J., Menke F., Talbot N.J. (2015). Septin-Dependent Assembly of the Exocyst Is Essential for Plant Infection by *Magnaporthe oryzae*. Plant Cell..

[17] Yang S., Zhou X., Guo P., Lin Y., Fan Q., Zuriegat Q., Lu S., Yang J., Yu W., Liu H. (2021). The Exocyst Regulates Hydrolytic Enzyme Secretion at Hyphal Tips and Septa in the Banana Fusarium Wilt Fungus Fusarium odoratissimum. Appl. Environ. Microbiol..

[18] Zeng J., Feng S., Wu B., Guo W. (2017). Polarized Exocytosis. Cold Spring Harb. Perspect. Biol..

[19] Lepore D.M., Martínez-Núñez L., Munson M. (2018). Exposing the Elusive Exocyst Structure. Trends Biochem. Sci..

[20] Boehm C., Field M.C. (2019). Evolution of late steps in exocytosis: Conservation and specialization of the exocyst complex. Wellcome Open Res..

[21] Žárský V. (2022). Exocyst functions in plants: Secretion and autophagy. FEBS Lett..

[22] Zajac A., Sun X., Zhang J., Guo W. (2005). Cyclical Regulation of the Exocyst and Cell Polarity Determinants for Polarized Cell Growth. Mol. Biol. Cell.

[23] Mei K., Guo W. (2018). The exocyst complex. Curr. Biol..

[24] Ganesan S.J., Feyder M.J., Chemmama I.E., Fang F., Rout M.P., Chait B.T., Shi Y., Munson M., Sali A. (2020). Integrative structure and function of the yeast exocyst complex. Protein Sci..

[25] Boehm C.M., Obado S., Gadelha C., Kaupisch A., Manna P.T., Gould G.W., Munson M., Chait B.T., Rout M.P., Field M.C. (2017). The Trypanosome Exocyst: A Conserved Structure Revealing a New Role in Endocytosis. PLoS Pathog..

[26] Liu J., Zhao Y., Sun Y., He B., Yang C., Svitkina T., Goldman Y.E., Guo W. (2012). Exo70 stimulates the Arp2/3 complex for lamellipodia formation and directional cell migration. Curr. Biol..

[27] Polgar N., Fogelgren B. (2017). Regulation of Cell Polarity by Exocyst-Mediated Trafficking. Cold Spring Harb. Perspect. Biol..

[28] TerBush D.R., Novick P. (1995). Sec6, Sec8, and Sec15 are components of a multisubunit complex which localizes to small bud tips in Saccharomyces cerevisiae. J. Cell Biol..

[29] Sharma K., Niraula P.M., Troell H.A., Adhikari M., Alshehri H.A., Alkharouf N.W., Lawrence K.S., Klink V.P. (2020). Exocyst components promote an incompatible interaction between Glycine max (soybean) and Heteroderaglycines (the soybean cyst nematode). Sci. Rep..

[30] Joseph-Strauss D., Zenvirth D., Simchen G., Barkai N. (2007). Spore germination in Saccharomyces cerevisiae: Global gene expression patterns and cell cycle landmarks. Genome Biol..

[31] Guo Q., Duan Y., Meng N., Liu Y., Luo G. (2020). The N-terminus of Sec3 is required for cell wall integrity in yeast. Biochimie.

[32] Guo Q., Meng N., Fan G., Sun D., Meng Y., Luo G., Liu Y. (2021). The role of the exocytic pathway in cell wall assembly in yeast. Yeast.

[33] Finger F.P., Novick P. (1997). Sec3p is involved in secretion and morphogenesis in Saccharomyces cerevisiae. Mol. Biol. Cell.

[34] Finger F.P., E Hughes T., Novick P. (1998). Sec3p Is a Spatial Landmark for Polarized Secretion in Budding Yeast. Cell.

[35] Howard R.J., Valent B. (1996). BREAKING AND ENTERING: Host Penetration by the Fungal Rice Blast Pathogen *Magnaporthe grisea*. Annu. Rev. Microbiol..

[36] Guan W., Feng J., Wang R., Ma Z., Wang W., Wang K., Zhu T. (2020). Functional analysis of the exocyst subunit BcExo70 in Botrytis cinerea. Curr. Genet..

[37] Heider M.R., Gu M., Duffy C.M., Mirza A.M., Marcotte L.L., Walls A.C., Farrall N., Hakhverdyan Z., Field M.C., Rout M.P. (2015). Subunit connectivity, assembly determinants and architecture of the yeast exocyst complex. Nat. Struct. Mol. Biol..

[38] Santos B., Snyder M. (2000). Sbe2p and Sbe22p, Two Homologous Golgi Proteins Involved in Yeast Cell Wall Formation. Mol. Biol. Cell.

[39] Fisher M.C., Gurr S.J., Cuomo C.A., Blehert D.S., Jin H., Stukenbrock E.H., Stajich J.E., Kahmann R., Boone C., Denning D.W. (2020). Threats Posed by the Fungal Kingdom to Humans, Wildlife, and Agriculture. mBio.

[40] Hsu S.C., E Ting A.E., Hazuka C.D., Davanger S., Kenny J.W., Kee Y., Scheller R.H. (1996). The mammalian brain rsec6/8 complex. Neuron.

[41] Guo W., Roth D., Gatti E., De Camilli P., Novick P. (1997). Identification and characterization of homologues of the Exocyst component Sec10p. FEBS Lett..

[42] Wiederkehr A., De Craene J.O., Ferro-Novick S., Novick P. (2004). Functional specialization within a vesicle tethering complex: Bypass of a subset of exocyst deletion mutants by Sec1p or Sec4p. J. Cell Biol..

[43] Guo P.P., Yong J.Y.A., Wang Y.M., Li C.R. (2016). Sec15 links bud site selection to polarised cell growth and exocytosis in Candida albicans. Sci. Rep..

[44] Salminen A., Novick P.J. (1989). The Sec15 protein responds to the function of the GTP binding protein, Sec4, to control vesicular traffic in yeast. J. Cell Biol..

[45] Boyd C., Hughes T., Pypaert M., Novick P. (2004). Vesicles carry most exocyst subunits to exocytic sites marked by the remaining two subunits, Sec3p and Exo70p. J. Cell Biol..

[46] Dong G., Hutagalung A.H., Fu C., Novick P., Reinisch K.M. (2005). The structures of exocyst subunit Exo70p and the Exo84p C-terminal domains reveal a common motif. Nat. Struct. Mol. Biol..

[47] Yamashita M., Kurokawa K., Sato Y., Yamagata A., Mimura H., Yoshikawa A., Sato K., Nakano A., Fukai S. (2010). Structural basis for the Rho- and phosphoinositide-dependent localization of the exocyst subunit Sec3. Nat. Struct. Mol. Biol..

[48] Pleskot R., Cwiklik L., Jungwirth P., Žárský V., Potocký M. (2015). Membrane targeting of the yeast exocyst complex. Biochim. Et Biophys. Acta (BBA) - Biomembr..

[49] Hsu S.C., TerBush D., Abraham M., Guo W. (2004). The exocyst complex in polarized exocytosis. Int. Rev. Cytol..

[50] Novick P., Garrett M.D., Brennwald P., Lauring A., Finger F.P., Collins R., TeeBush D. (1995). Control of exocytosis in yeast. Cold Spring Harb. Symp. Quant. Biol..

[51] He B., Xi F., Zhang X., Zhang J., Guo W. (2007). Exo70 interacts with phospholipids and mediates the targeting of the exocyst to the plasma membrane. EMBO J..

[52] Ungermann C., Kümmel D. (2019). Structure of membrane tethers and their role in fusion. Traffic.

[53] Santana-Molina C., Gutierrez F., Devos D.P. (2021). Homology and Modular Evolution of CATCHR at the Origin of the Eukaryotic Endomembrane System. Genome Biol. Evol..

[54] Ahmed S.M., Nishida-Fukuda H., Li Y., McDonald W.H., Gradinaru C.C., Macara I.G. (2018). Exocyst dynamics during vesicle tethering and fusion. Nat. Commun..

[55] Pereira C., Stalder D., Anderson G.S., Shun-Shion A.S., Houghton J., Antrobus R., Chapman M.A., Fazakerley D.J., Gershlick D.C. (2023). The exocyst complex is an essential component of the mammalian constitutive secretory pathway. J. Cell Biol..

[56] Picco A., Irastorza-Azcarate I., Specht T., Böke D., Pazos I., Rivier-Cordey A.-S., Devos D.P., Kaksonen M., Gallego O. (2017). The In Vivo Architecture of the Exocyst Provides Structural Basis for Exocytosis. Cell.

[57] Hernandez A.C., Ortiz S., I Betancur L., Dojčilović R., Picco A., Kaksonen M., Oliva B., Gallego O. (2024). PyF2F: A robust and simplified fluorophore-to-fluorophore distance measurement tool for Protein interactions from Imaging Complexes after Translocation experiments. NAR Genom. Bioinform..

[58] Forson J., Martínez-Núñez L., Lepore D., Munson M. (2020). Binding of the exocyst complex to the SNARE regulator Sec1. FASEB J..

[59] Morgera F., Sallah M.R., Dubuke M.L., Gandhi P., Brewer D.N., Carr C.M., Munson M. (2012). Regulation of exocytosis by the exocyst subunit Sec6 and the SM protein Sec1. Mol. Biol. Cell.

[60] Miller B.K., Rossi G., Hudson S., Cully D., Baker R.W., Brennwald P. (2023). Allosteric regulation of exocyst: Discrete activation of tethering by two spatial signals. J. Cell Biol..

[61] Yue P., Zhang Y., Mei K., Wang S., Lesigang J., Zhu Y., Dong G., Guo W. (2017). Sec3 promotes the initial binary t-SNARE complex assembly and membrane fusion. Nat. Commun..

[62] Guo Q., Zhang T., Meng N., Duan Y., Meng Y., Sun D., Liu Y., Luo G. (2020). Sphingolipids are required for exocyst polarity and exocytic secretion in Saccharomyces cerevisiae. Cell Biosci..

[63] Tsuji K., Kitade Y., Sumita T., Tanaka C. (2021). An exocyst component, Sec5, is essential for ascospore formation in Bipolaris maydis. Mycoscience.

[64] Panepinto J., Komperda K., Frases S., Park Y., Djordjevic J.T., Casadevall A., Williamson P.R. (2009). Sec6-dependent sorting of fungal extracellular exosomes and laccase of *Cryptococcus neoformans*. Mol. Microbiol..

[65] Songer J.A., Munson M. (2009). Sec6p Anchors the Assembled Exocyst Complex at Sites of Secretion. Mol. Biol. Cell.

[66] Roth D., Guo W., Novick P. (1998). Dominant Negative Alleles of *SEC10* Reveal Distinct Domains Involved in Secretion and Morphogenesis in Yeast. Mol. Biol. Cell.

[67] van Gisbergen P.A., Wu S.-Z., Chang M., Pattavina K.A., Bartlett M.E., Bezanilla M. (2018). An ancient Sec10–formin fusion provides insights into actin-mediated regulation of exocytosis. J. Cell Biol..

[68] Wu S., Mehta S.Q., Pichaud F., Bellen H.J., Quiocho F.A. (2005). Sec15 interacts with Rab11 via a novel domain and affects Rab11 localization in vivo. Nat. Struct. Mol. Biol..

[69] Zhu Y., Wu B., Guo W. (2017). The role of Exo70 in exocytosis and beyond. Small GTPases.

[70] Guo W., Grant A., Novick P. (1999). Exo84p Is an Exocyst Protein Essential for Secretion. J. Biol. Chem..

[71] Liu J., Zuo X., Yue P., Guo W. (2007). Phosphatidylinositol 4,5-Bisphosphate Mediates the Targeting of the Exocyst to the Plasma Membrane for Exocytosis in Mammalian Cells. Mol. Biol. Cell.

[72] Zhang X., Orlando K., He B., Xi F., Zhang J., Zajac A., Guo W. (2008). Membrane association and functional regulation of Sec3 by phospholipids and Cdc42. J. Cell Biol..

[73] Inoue M., Akama T., Jiang Y., Chun T.-H. (2015). The Exocyst Complex Regulates Free Fatty Acid Uptake by Adipocytes. PLoS ONE.

[74] Hsu S.C., Hazuka C.D., Roth R., Foletti D.L., Heuser J., Scheller R.H. (1998). Subunit composition, protein interactions, and structures of the mammalian brain sec6/8 complex and septin filaments. Neuron.

[75] Hutagalung A.H., Coleman J., Pypaert M., Novick P.J. (2009). An internal domain of Exo70p is required for actin-independent localization and mediates assembly of specific exocyst components. Mol. Biol. Cell..

[76] Zhang X.-M., Ellis S., Sriratana A., Mitchell C.A., Rowe T. (2004). Sec15 Is an Effector for the Rab11 GTPase in Mammalian Cells. J. Biol. Chem..

[77] Zhang X., Bi E., Novick P., Du L., Kozminski K.G., Lipschutz J.H., Guo W. (2001). Cdc42 Interacts with the Exocyst and Regulates Polarized Secretion. J. Biol. Chem..

[78] Luo G., Zhang J., Guo W. (2014). The role of Sec3p in secretory vesicle targeting and exocyst complex assembly. Mol. Biol. Cell.

[79] Yan X., Tang B., Ryder L.S., MacLean D., Were V.M., Eseola A.B., Cruz-Mireles N., Ma W., Foster A.J., Osés-Ruiz M. (2023). The transcriptional landscape of plant infection by the rice blast fungus *Magnaporthe oryzae* reveals distinct families of temporally co-regulated and structurally conserved effectors. Plant Cell.

[80] Guo W., Tamanoi F., Novick P. (2001). Spatial regulation of the exocyst complex by Rho1 GTPase. Nat. Cell Biol..

[81] Wu H., Turner C., Gardner J., Temple B., Brennwald P. (2010). The Exo70 Subunit of the Exocyst Is an Effector for Both Cdc42 and Rho3 Function in Polarized Exocytosis. Mol. Biol. Cell.

[82] Hashizume K., Cheng Y.-S., Hutton J.L., Chiu C.-H., Carr C.M. (2009). Yeast Sec1p Functions before and after Vesicle Docking. Mol. Biol. Cell.

[83] Dobbelaere J., Barral Y. (2004). Spatial Coordination of Cytokinetic Events by Compartmentalization of the Cell Cortex. Science.

[84] Pruyne D., Legesse-Miller A., Gao L., Dong Y., Bretscher A. (2004). Mechanisms of Polarized Growth And Organelle Segregation in Yeast. Annu. Rev. Cell Dev. Biol..

[85] VerPlank L., Li R. (2005). Cell Cycle-regulated Trafficking of Chs2 Controls Actomyosin Ring Stability during Cytokinesis. Mol. Biol. Cell.

[86] Martin-Urdiroz M., Deeks M.J., Horton C.G., Dawe H.R., Jourdain I. (2016). The Exocyst Complex in Health and Disease. Front. Cell Dev. Biol..

[87] Koon A.C., Chen Z.S., Peng S., Fung J.M.S., Zhang X., Lembke K.M., Chow H.K., Frank C.A., Jiang L., Lau K.-F. (2018). *Drosophila* Exo70 Is Essential for Neurite Extension and Survival under Thermal Stress. J. Neurosci..

[88] Saeed B., Brillada C., Trujillo M. (2019). Dissecting the plant exocyst. Curr. Opin. Plant Biol..

[89] Phanprasert Y., Maciszewski K., Gentekaki E., Dacks J.B. (2023). Comparative genomic analysis illustrates evolutionary dynamics of multisubunit tethering complexes across green algal diversity. J. Eukaryot. Microbiol..

[90] Synek L., Schlager N., Eliáš M., Quentin M., Hauser M., Žárský V. (2006). AtEXO70A1, a member of a family of putative exocyst subunits specifically expanded in land plants, is important for polar growth and plant development. Plant J..

[91] Zhang Y., Liu C.M., Emons A.M., Ketelaar T. (2010). The plant exocyst. J. Integr. Plant Biol..

[92] Taheri-Talesh N., Horio T., Araujo-Bazán L., Dou X., Espeso E.A., Peñalva M.A., Osmani S.A., Oakley B.R. (2008). The Tip Growth Apparatus of *Aspergillus nidulans*. Mol. Biol. Cell.

[93] Jones L.A., Sudbery P.E. (2010). Spitzenkörper, Exocyst, and Polarisome Components in Candida albicans Hyphae Show Different Patterns of Localization and Have Distinct Dynamic Properties. Eukaryot. Cell.

[94] Khalaj V., Brookman J.L., Robson G.D. (2001). A Study of the Protein Secretory Pathway of Aspergillus niger Using a Glucoamylase–GFP Fusion Protein. Fungal Genet. Biol..

[95] Grindstaff K.K., Yeaman C., Anandasabapathy N., Hsu S.-C., Rodriguez-Boulan E., Scheller R.H., Nelson W. (1998). Sec6/8 complex is recruited to cell-cell contacts and specifies transport vesicle delivery to the basal-lateral membrane in epithelial cells. Cell.

[96] Matern H.T., Yeaman C., Nelson W.J., Scheller R.H. (2001). The Sec6/8 complex in mammalian cells: Characterization of mammalian Sec3, subunit interactions, and expression of subunits in polarized cells. Proc. Natl. Acad. Sci. USA.

[97] Stinchcombe J.C., Bossi G., Booth S., Griffiths G.M. (2001). The Immunological Synapse of CTL Contains a Secretory Domain and Membrane Bridges. Immunity.

[98] Fernandez-Calvino L., Faulkner C., Walshaw J., Saalbach G., Bayer E., Benitez-Alfonso Y., Maule A. (2011). Arabidopsis Plasmodesmal Proteome. PLoS ONE.

[99] Caballero-Lima D., Sudbery P.E. (2014). In *Candida albicans*, phosphorylation of Exo84 by Cdk1-Hgc1 is necessary for efficient hyphal extension. Mol. Biol. Cell.

[100] Luo G., Zhang J., Luca F.C., Guo W. (2013). Mitotic phosphorylation of Exo84 disrupts exocyst assembly and arrests cell growth. J. Cell Biol..

[101] Duan Y., Guo Q., Zhang T., Meng Y., Sun D., Luo G., Liu Y. (2019). Cyclin-dependent kinase–mediated phosphorylation of the exocyst subunit Exo84 in late G1 phase suppresses exocytic secretion and cell growth in yeast. J. Biol. Chem..

[102] Caza M., Kronstad J.W. (2019). The cAMP/Protein Kinase A Pathway Regulates Virulence and Adaptation to Host Conditions in Cryptococcus neoformans. Front. Cell. Infect. Microbiol..

[103] González B., Cullen P.J. (2022). Regulation of Cdc42 protein turnover modulates the filamentous growth MAPK pathway. J. Cell Biol..

[104] Adamo J.E., Moskow J.J., Gladfelter A.S., Viterbo D., Lew D.J., Brennwald P.J. (2001). Yeast Cdc42 functions at a late step in exocytosis, specifically during polarized growth of the emerging bud. J. Cell Biol..

[105] Bendezú F.O., Vincenzetti V., Martin S.G. (2012). Fission Yeast Sec3 and Exo70 Are Transported on Actin Cables and Localize the Exocyst Complex to Cell Poles. PLoS ONE.

[106] Tay Y.D., Leda M., Spanos C., Rappsilber J., Goryachev A.B., Sawin K.E. (2019). Fission Yeast NDR/LATS Kinase Orb6 Regulates Exocytosis via Phosphorylation of the Exocyst Complex. Cell Rep..

[107] Lipschutz J.H., Lingappa V.R., Mostov K.E. (2003). The Exocyst Affects Protein Synthesis by Acting on the Translocation Machinery of the Endoplasmic Reticulum. J. Biol. Chem..

[108] Lipatova Z., Tokarev A.A., Jin Y., Mulholland J., Weisman L.S., Segev N. (2008). Direct interaction between a myosin V motor and the Rab GTPases Ypt31/32 is required for polarized secretion. Mol. Biol. Cell..

[109] Munson M., Novick P. (2006). The exocyst defrocked, a framework of rods revealed. Nat. Struct. Mol. Biol..

[110] Sudbery P. (2011). Fluorescent proteins illuminate the structure and function of the hyphal tip apparatus. Fungal Genet. Biol..

[111] Marks B., Stowell M.H.B., Vallis Y., Mills I.G., Gibson A., Hopkins C.R., McMahon H.T. (2001). GTPase activity of dynamin and resulting conformation change are essential for endocytosis. Nature.

[112] Takeshita N., Higashitsuji Y., Konzack S., Fischer R. (2008). Apical Sterol-rich Membranes Are Essential for Localizing Cell End Markers That Determine Growth Directionality in the Filamentous Fungus *Aspergillus nidulans*. Mol. Biol. Cell.

[113] Goyal A., Takaine M., Simanis V., Nakano K. (2011). Dividing the spoils of growth and the cell cycle: The fission yeast as a model for the study of cytokinesis. Cytoskeleton.

[114] Wu H., Rossi G., Brennwald P. (2008). The ghost in the machine: Small GTPases as spatial regulators of exocytosis. Trends Cell Biol..

[115] Shen D., Yuan H., Hutagalung A., Verma A., Kümmel D., Wu X., Reinisch K., McNew J.A., Novick P. (2013). The synaptobrevin homologue Snc2p recruits the exocyst to secretory vesicles by binding to Sec6p. J. Cell Biol..

[116] Douglas L.M., Konopka J.B. (2016). Plasma membrane organization promotes virulence of the human fungal pathogen Candida albicans. J. Microbiol..

[117] Bassilana M., Puerner C., Arkowitz R.A. (2020). External signal–mediated polarized growth in fungi. Curr. Opin. Cell Biol..

[118] Pfeffer S.R. (1999). Transport-vesicle targeting: Tethers before SNAREs. Nat. Cell Biol..

[119] Cai H., Reinisch K., Ferro-Novick S. (2007). Coats, Tethers, Rabs, and SNAREs Work Together to Mediate the Intracellular Destination of a Transport Vesicle. Dev. Cell.

[120] Bröcker C., Engelbrecht-Vandré S., Ungermann C. (2010). Multisubunit Tethering Complexes and Their Role in Membrane Fusion. Curr. Biol..

[121] Donovan K.W., Bretscher A. (2015). Tracking individual secretory vesicles during exocytosis reveals an ordered and regulated process. J. Cell Biol..

[122] Nicholson K.L., Munson M., Miller R.B., Filip T.J., Fairman R., Hughson F.M. (1998). Regulation of SNARE complex assembly by an N-terminal domain of the t-SNARE Sso1p. Nat. Struct. Mol. Biol..

[123] Hughson F.M., Munson M., Chen X., Cocina A.E., Schultz S.M. (2000). Interactions within the yeast t-SNARE Sso1p that control SNARE complex assembly. Nat. Struct. Mol. Biol..

[124] Dubuke M.L., Maniatis S., Shaffer S.A., Munson M. (2015). The Exocyst Subunit Sec6 Interacts with Assembled Exocytic SNARE Complexes. J. Biol. Chem..

[125] Zhang X., Zajac A., Zhang J., Wang P., Li M., Murray J., TerBush D., Guo W. (2005). The Critical Role of Exo84p in the Organization and Polarized Localization of the Exocyst Complex. J. Biol. Chem..

[126] Rivera-Molina F., Toomre D. (2013). Live-cell imaging of exocyst links its spatiotemporal dynamics to various stages of vesicle fusion. J. Cell Biol..

[127] Rossi G., Lepore D., Kenner L., Czuchra A.B., Plooster M., Frost A., Munson M., Brennwald P. (2020). Exocyst structural changes associated with activation of tethering downstream of Rho/Cdc42 GTPases. J. Cell Biol..

[128] Rossi G., Puller G.C., Brennwald P. (2024). Asymmetric tethering by exocyst in vitro requires a Rab GTPase, an R-SNARE and a Sac1-sensitive phosphoinositide lipid. Mol. Biol. Cell.

[129] Hattendorf D.A., Andreeva A., Gangar A., Brennwald P.J., Weis W.I. (2007). Structure of the yeast polarity protein Sro7 reveals a SNARE regulatory mechanism. Nature.

[130] Baek K., Knödler A., Lee S.H., Zhang X., Orlando K., Zhang J., Foskett T.J., Guo W., Dominguez R. (2010). Structure-Function Study of the N-terminal Domain of Exocyst Subunit Sec3. J. Biol. Chem..

[131] Jin Y., Sultana A., Gandhi P., Franklin E., Hamamoto S., Khan A.R., Munson M., Schekman R., Weisman L.S. (2011). Myosin V Transports Secretory Vesicles via a Rab GTPase Cascade and Interaction with the Exocyst Complex. Dev. Cell.

[132] Sánchez-León E., Verdín J., Freitag M., Roberson R.W., Bartnicki-Garcia S., Riquelme M. (2011). Traffic of Chitin Synthase 1 (CHS-1) to the Spitzenkörper and Developing Septa in Hyphae of Neurospora crassa: Actin Dependence and Evidence of Distinct Microvesicle Populations. Eukaryot. Cell.

[133] Herrero S., Takeshita N., Fischer R. (2014). F-Box Protein RcyA Controls Turnover of the Kinesin-7 Motor KipA in Aspergillus nidulans. Eukaryot. Cell.

[134] Braaksma M., Smilde A.K., van der Werf M.J., Punt P.J. (2009). The effect of environmental conditions on extracellular protease activity in controlled fermentations of Aspergillus niger. Microbiology.

[135] Mei K., Li Y., Wang S., Shao G., Wang J., Ding Y., Luo G., Yue P., Liu J.J., Wang X. (2018). Cryo-EM structure of the exocyst complex. Nat. Struct. Mo. Biol..

[136] Togneri J., Cheng Y.-S., Munson M., Hughson F.M., Carr C.M. (2006). Specific SNARE complex binding mode of the Sec1/Munc-18 protein, Sec1p. Proc. Natl. Acad. Sci. USA.

[137] Wang H., Tang X., Balasubramanian M.K. (2003). Rho3p regulates cell separation by modulating exocyst function in Schizosaccharomyces pombe. Genetics.

[138] Pérez P., Portales E., Santos B. (2015). Rho4 interaction with exocyst and septins regulates cell separation in fission yeast. Microbiology.

[139] France Y.E., Boyd C., Coleman J., Novick P.J. (2006). The polarity-establishment component Bem1p interacts with the exocyst complex through the Sec15p subunit. J. Cell Sci..

[140] Zanolari B., Rockenbauch U., Trautwein M., Clay L., Barral Y., Spang A. (2011). Transport to the plasma membrane is regulated differently early and late in the cell cycle in *Saccharomyces cerevisiae*. J. Cell Sci..

[141] Gingras R.M., Lwin K.M., Miller A.M., Bretscher A. (2020). Yeast Rgd3 is a phospho-regulated F-BAR-containing RhoGAP involved in the regulation of Rho3 distribution and cell morphology. Mol. Biol. Cell.

[142] Desai J.V. (2018). *Candida albicans* Hyphae: From Growth Initiation to Invasion. J. Fungi.

[143] Rollenhagen C., Mamtani S., Ma D., Dixit R., Eszterhas S., Lee S.A. (2020). The Role of Secretory Pathways in *Candida albicans* Pathogenesis. J. Fungi.

[144] Li C., Chen P., Vaughan J., Lee K.F., Vale W. (2007). Urocortin 3 regulates glucose-stimulated insulin secretion and energy homeostasis. Proc. Natl. Acad. Sci. USA.

[145] Martín-Cuadrado A.B., Morrell J.L., Konomi M., An H., Petit C., Osumi M., Balasubramanian M., Gould K.L., del Rey F., de Aldana C.R.V. (2005). Role of Septins and the Exocyst Complex in the Function of Hydrolytic Enzymes Responsible for Fission Yeast Cell Separation. Mol. Biol. Cell.

[146] Wang H., Eto M., Steers W.D., Somlyo A.P., Somlyo A.V. (2002). RhoA-mediated Ca2+ sensitization in erectile function. J. Biol. Chem..

[147] Naranjo-Ortiz M.Á., Gabaldón T. (2019). Fungal evolution: Diversity, taxonomy and phylogeny of the Fungi. Biol. Rev..

[148] Pukkila-Worley R., Gerrald Q.D., Kraus P.R., Boily M.-J., Davis M.J., Giles S.S., Cox G.M., Heitman J., Alspaugh J.A. (2005). Transcriptional Network of Multiple Capsule and Melanin Genes Governed by the *Cryptococcus neoformans* Cyclic AMP Cascade. Eukaryot. Cell.

[149] Barelle C.J., Priest C.L., MacCallum D.M., Gow N.A.R., Odds F.C., Brown A.J.P. (2006). Niche-specific regulation of central metabolic pathways in a fungal pathogen. Cell. Microbiol..

[150] Ivanov S., Fedorova E.E., Limpens E., De Mita S., Genre A., Bonfante P., Bisseling T. (2012). Rhizobium-legume symbiosis shares an exocytotic pathway required for arbuscule formation. Proc. Natl. Acad. Sci. USA.

[151] Genre A., Ivanov S., Fendrych M., Faccio A., Žárský V., Bisseling T., Bonfante P. (2011). Multiple Exocytotic Markers Accumulate at the Sites of Perifungal Membrane Biogenesis in Arbuscular Mycorrhizas. Plant Cell Physiol..

[152] Caballero-Lima D., Kaneva I.N., Watton S.P., Sudbery P.E., Craven C.J. (2013). The Spatial Distribution of the Exocyst and Actin Cortical Patches Is Sufficient To Organize Hyphal Tip Growth. Eukaryot. Cell.

[153] Heasley L.R., Garcia G., McMurray M.A. (2014). Off-target effects of the septin drug forchlorfenuron on nonplant eukaryotes. Eukaryot. Cell..

[154] Heasley L.R., Singer E., Cooperman B.J., McMurray M.A. (2020). *Saccharomyces* spores are born prepolarized to outgrow away from spore–spore connections and penetrate the ascus wall. Yeast.

[155] Dagley M.J., Gentle I.E., Beilharz T.H., Pettolino F.A., Djordjevic J.T., Lo T.L., Uwamahoro N., Rupasinghe T., Tull D.L., McConville M. (2010). Cell wall integrity is linked to mitochondria and phospholipid homeostasis in *Candida albicans* through the activity of the post-transcriptional regulator Ccr4-Pop2. Mol. Microbiol..

[156] Ryder L.S., Dagdas Y.F., Kershaw M.J., Venkataraman C., Madzvamuse A., Yan X., Cruz-Mireles N., Soanes D.M., Oses-Ruiz M., Styles V. (2019). A sensor kinase controls turgor-driven plant infection by the rice blast fungus. Nature.

[157] Grote E., Carr C.M., Novick P.J. (2000). Ordering the Final Events in Yeast Exocytosis. J. Cell Biol..

[158] Gómez-Sánchez R., Rose J., Guimarães R., Mari M., Papinski D., Rieter E., Geerts W.J., Hardenberg R., Kraft C., Ungermann C. (2018). Atg9 establishes Atg2-dependent contact sites between the endoplasmic reticulum and phagophores. J. Cell Biol..

[159] Geng J., Klionsky D.J. (2010). Determining Atg protein stoichiometry at the phagophore assembly site by fluorescence microscopy. Autophagy.

[160] Nair U., Thumm M., Klionsky D.J., Krick R. (2011). GFP-Atg8 protease protection as a tool to monitor autophagosome biogenesis. Autophagy.

[161] Hamasaki M., Noda T., Ohsumi Y. (2003). The Early Secretory Pathway Contributes to Autophagy in Yeast. Cell Struct. Funct..

[162] Singh S., Kumari R., Chinchwadkar S., Aher A., Matheshwaran S., Manjithaya R. (2019). Exocyst Subcomplex Functions in Autophagosome Biogenesis by Regulating Atg9 Trafficking. J. Mol. Biol..

[163] Sharifmoghadam M.R., De Leon N., Hoya M., Curto M., Valdivieso M.-H. (2010). Different steps of sexual development are differentially regulated by the Sec8p and Exo70p exocyst subunits. FEMS Microbiol. Lett..

[164] Imada K., Nakamura T., Nakamura-Kubo M., Hirata A., Shimoda C., Brennwald M.E.P., Walter M.E.P., Li W.-Z., Yu Z.-Y., Ma P.-F. (2016). The exocytic Rabs Ypt3 and Ypt2 regulate the early step of biogenesis of the spore plasma membrane in fission yeast. Mol. Biol. Cell.

[165] Kim D.-U., Hayles J., Kim D., Wood V., Park H.-O., Won M., Yoo H.-S., Duhig T., Nam M., Palmer G. (2010). Analysis of a genome-wide set of gene deletions in the fission yeast Schizosaccharomyces pombe. Nat. Biotechnol..

[166] Giraldo M.C., Dagdas Y.F., Gupta Y.K., Mentlak T.A., Yi M., Martinez-Rocha A.L., Saitoh H., Terauchi R., Talbot N.J., Valent B. (2013). Two distinct secretion systems facilitate tissue invasion by the rice blast fungus *Magnaporthe oryzae*. Nat. Commun..

[167] Kwon M.J., Arentshorst M., Fiedler M., de Groen F.L.M., Punt P.J., Meyer V., Ram A.F.J. (2014). Molecular genetic analysis of vesicular transport in Aspergillus niger reveals partial conservation of the molecular mechanism of exocytosis in fungi. Microbiology.

[168] Bowman S.M., Free S.J. (2006). The structure and synthesis of the fungal cell wall. BioEssays.

[169] Khang C.H., Berruyer R., Giraldo M.C., Kankanala P., Park S.-Y., Czymmek K., Kang S., Valent B. (2010). Translocation of *Magnaporthe oryzae* Effectors into Rice Cells and Their Subsequent Cell-to-Cell Movement. Plant Cell.

[170] Takemoto D., Kamakura S., Saikia S., Becker Y., Wrenn R., Tanaka A., Sumimoto H., Scott B. (2011). Polarity proteins Bem1 and Cdc24 are components of the filamentous fungal NADPH oxidase complex. Proc. Natl. Acad. Sci. USA.

[171] Fendrych M., Synek L., Pečenková T., Drdová E.J., Sekereš J., de Rycke R., Nowack M.K., Žárský V. (2013). Visualization of the exocyst complex dynamics at the plasma membrane of *Arabidopsis thaliana*. Mol. Biol. Cell.

[172] Lo Presti L., Lanver D., Schweizer G., Tanaka S., Liang L., Tollot M., Zuccaro A., Reissmann S., Kahmann R. (2015). Fungal Effectors and Plant Susceptibility. Annu. Rev. Plant Biol..

[173] Nobile C.J., Johnson A.D. (2015). *Candida albicans* Biofilms and Human Disease. Annu. Rev. Microbiol..

[174] Latgé J.-P., Chamilos G. (2019). Aspergillus fumigatus and Aspergillosis in 2019. Clin. Microbiol. Rev..

[175] Heilmann C. (2011). Adhesion Mechanisms of Staphylococci. Adv. Exp. Med. Biol..

[176] Zhou J., Chai X., Zhang L., George T.S., Wang F., Feng G. (2020). Different Arbuscular Mycorrhizal Fungi Cocolonizing on a Single Plant Root System Recruit Distinct Microbiomes. mSystems.

[177] Beck M., Zhou J., Faulkner C., MacLean D., Robatzek S. (2012). Spatio-Temporal Cellular Dynamics of the *Arabidopsis* Flagellin Receptor Reveal Activation Status-Dependent Endosomal Sorting. Plant Cell.

[178] Moskalenko S., Henry D.O., Rosse C., Mirey G., Camonis J.H., White M.A. (2001). The exocyst is a Ral effector complex. Nat. Cell Biol..

[179] Li L., Zhu X.-M., Su Z.-Z., Del Poeta M., Liu X.-H., Lin F.-C. (2021). Insights of roles played by septins in pathogenic fungi. Virulence.

